# A Prediction Model for Risk of Death in Kidney Transplant Recipients

**DOI:** 10.1001/jamanetworkopen.2026.7452

**Published:** 2026-04-23

**Authors:** Charlotte Debiais-Deschamps, Marc Raynaud, Agathe Truchot, Yannis Lombardi, Gillian Divard, Vivek A. Rudrapatna, Soufian Meziyerh, Jesper Kers, Danny van der Helm, Aiko P. J. de Vries, Juliette Gueguen, Arnaud Del Bello, Elisabet Van Loon, Antoine Bouquegneau, Christophe Legendre, Xavier Jouven, Adam Mussell, Brian McCauley, Sabrina Emms, Shristi Mapchan, Caroline C. Jadlowiec, Raymond L. Heilman, Devika Das, Nassim Kamar, Dany Anglicheau, Kevin J. Fowler, Maarten Naesens, Carmen Lefaucheur, Olivier Aubert, Peter P. Reese, Alexandre Loupy

**Affiliations:** 1 Paris Institute for Transplantation and Organ Regeneration, Institut National de la Santé et de la Recherche Médicale U970, Université Paris Cité, Paris, France; 2 Kidney Transplant Department, Necker Hospital, Assistance Publique-Hôpitaux de Paris, Paris, France; 3 Department of Nephrology and Renal Transplantation, Hôpital Tenon, Assistance Publique-Hôpitaux de Paris, Paris, France; 4 Kidney Transplant Department, Saint-Louis Hospital, Assistance Publique-Hôpitaux de Paris, Paris, France; 5 Division of Gastroenterology and Hepatology, Department of Medicine, University of California, San Francisco; 6 Bakar Computational Health Sciences Institute, University of California, San Francisco; 7 Leiden Transplant Center, Leiden University Medical Center, Leiden University, Leiden, the Netherlands; 8 Division of Nephrology, Department of Internal Medicine, Leiden University Medical Center, Leiden University, Leiden, the Netherlands; 9 Department of Nephrology and Clinical Immunology, University Hospital of Tours, Tours, France; 10 Université François Rabelais, Tours, France; 11 Department of Nephrology and Organ Transplantation, Toulouse University Hospital, Institut National de la Santé et de la Recherche Médicale Unité Mixte de Recherche 1291, Toulouse Institute for Infectious and Inflammatory Diseases, University Paul Sabatier, Toulouse, France; 12 Department of Nephrology and Renal Transplantation, University Hospitals Leuven, Leuven, Belgium; 13 Division of Nephrology, University Hospital Liège, University of Liège, Liège, Belgium; 14 Renal-Electrolyte and Hypertension Division, Department of Medicine, Perelman School of Medicine at the University of Pennsylvania, Philadelphia; 15 Penn Transplant Institute, Hospital of the University of Pennsylvania, Philadelphia; 16 Department of Biostatistics, Epidemiology and Informatics, Perelman School of Medicine at the University of Pennsylvania, Philadelphia; 17 Department of Medicine, Mayo Clinic, Phoenix, Arizona; 18 The Voice of The Patient Inc, St Louis, Missouri; 19 Université Paris Cité, Institut National de la Santé et de la Recherche Médicale U1151, Centre National de la Recherche Scientifique Unités Mixtes de Recherche 8253, Institut Necker Enfants Malades, Paris, France

## Abstract

**Importance:**

Accurate prediction of patient mortality after kidney transplant is an unmet need.

**Objective:**

To develop and validate an integrative prediction model that predicts short- and long-term patient mortality for kidney recipients.

**Design, Setting, and Participants:**

This international cohort study included patients who underwent transplants between 2004 and 2023 from 14 academic medical centers from Europe and the US. The derivation cohort consisted of 1566 consecutive adult kidney recipients in a deeply phenotyped cohort prospectively recruited in 1 French center between 2004 and 2014. External validation cohorts consisted of 10 951 kidney recipients from 7 centers in France, 3 centers in Europe, and 3 centers in North America who underwent kidney transplants between 2006 and 2023. Data were analyzed from January 2023 to June 2025.

**Main Outcome and Measures:**

All-cause mortality was assessed, and 121 candidate prognostic factors, comprising demographics and clinical, biological, imaging, and immunological parameters, were collected.

**Results:**

Among 12 517 kidney transplant recipients, including 1566 in the derivation cohort (mean [SD] age, 50.05 [14.31] years; 942 male [60.15%]) and 10 951 in validation cohorts (mean [SD] age, 53.32 [13.97] years; 6766 male [61.78%]), 2486 patients (19.9%) died after a median (IQR) follow-up of 5.08 (2.97-7.00) years. Fourteen prognostic factors (including clinical, biological, and imaging risk factors) were independently associated with patient death (eg, patient age: hazard ratio per 1-year increase in age, 1.07 [95% CI, 1.06-1.08]; *P* < .001) and were combined into a risk prediction model (mBox). The model exhibited accurate calibration and discrimination in the derivation cohort, with C statistics of 0.82 (95% CI, 0.77-0.87) and 0.80 (95% CI, 0.78-0.82) at 1 and 10 years, respectively, after the transplant. Abbreviated models were developed and validated to ensure model generalizability. Performance of abbreviated models was confirmed in external validation cohorts from France (C statistic, 0.76 [95% CI, 0.73-0.78]), Europe (C statistic, 0.74 [95% CI, 0.72-0.76]), the US (C statistic, 0.74 [95% CI, 0.70-0.78]), the Greater Paris University Hospital database (C statistic, 0.79 [95% CI, 0.77-0.81]), and the University of California at San Francisco database (C statistic, 0.70 [95% CI, 0.65-0.74]). The model was also validated in a series of subpopulations (eg, women vs men: C statistic, 0.81 [95% CI, 0.77-0.84] vs 0.79 [95% CI, 0.77-0.82]) and clinical scenarios (eg, before COVID-19 era: C statistic, 0.79 [95% CI, 0.77-0.81]) with good and stable performance.

**Conclusions and Relevance:**

In this study, an accurate prediction model for mortality among kidney recipients, computable at the time of transplant, was developed and externally validated. Results suggest that this model may help stratify patient risk of death, allowing for improved medical decisions.

## Key Points

**Question:**

What is the accuracy of a novel model for predicting death among kidney transplant recipients?

**Findings:**

In this prognostic study that included 12 517 kidney recipients and 121 candidate risk factors, 14 prognostic factors were independently associated with death and were combined into a risk prediction model, which exhibited accurate calibration and discrimination. Performance was confirmed in external validation cohorts from France, Europe, the US, and clinical data warehouses.

**Meaning:**

The risk prediction model developed in this study is the first model based on a deeply phenotyped cohort, demonstrated high prediction performances, and was validated in multiple subpopulations, clinical scenarios, centers, and countries.

## Teaser

This prognostic study investigates the accuracy of a mortality prediction model among patients who received kidney transplants.

## Introduction

The number of individuals with end-stage chronic kidney disease worldwide has increased over time, exceeding 7 million patients in 2020.^1^ For individuals with this disease, kidney transplant is the best treatment in terms of patient survival^1,2,3,4,5^ and quality of life^6,7^ and cost-effectiveness^8,9^ compared with dialysis, even in comorbid or elderly populations.^10,11^ Although the number of kidney transplants performed each year has increased, it follows a slower pace than the increase of individuals on the waiting list, resulting in an organ shortage.^12,13^

In this context, a kidney recipient death prediction model may allow identification of patients at high risk, with associated improvements in transplant clinical practice, allowing for the ability to evaluate the individual risk of posttransplant mortality before transplant surgery, thereby guiding decision-making. However, developing such a model is difficult given that death after kidney transplant depends on many parameters, such as donor age, history, or cause of death^14,15,16,17^; imaging parameters^18,19^; patient medical history (eg, diabetes, dialysis duration, and hypertension^15,16,17,20,21,22,23,24^); biological parameters^16,21^; and allograft-related parameters, such as human leukocyte antigen mismatches or cold ischemia time.^15,16,24^

According to a literature review we conducted (eMethods 1 in Supplement 1), studies that developed kidney recipient death prediction models in the past 20 years had methodological shortcomings. These studies rarely considered key potential risk factors associated with patient death and commonly relied on registry data^15,20^ in which comorbid conditions were frequently underreported^25^ and biological parameters were frequently missing.^15,22^ In addition, these studies also frequently lacked external validation, and the follow-up duration was too brief for long-term prediction.^13,14,21,22,23^ The goal of this study was therefore to identify predictors of death after kidney transplant and to develop and validate a prediction model that would help stratify patient risk of death and optimize posttransplant patient treatment. This was done using a large, international, highly phenotyped cohort of kidney recipients with extensive data collection and long-term follow-up.

## Methods

### Study Design and Population

A total of 12 517 patients were included in this prognostic study. This population was divided into 1566 patients in the derivation cohort and 10 951 patients in validation cohorts. The study is reported following the Transparent Reporting of a Multivariable Prediction Model for Individual Prognosis or Diagnosis (TRIPOD) reporting guidelines.

#### Derivation Cohort

The derivation cohort comprised 1566 consecutive patients aged 18 years or older who were prospectively enrolled at the time of transplant of a kidney from a living or deceased donor at Necker Hospital in France between January 1, 2004, and January 1, 2014. We excluded patients who underwent multiorgan transplant (see study flowchart in eMethods 2 in Supplement 1).

Clinical data were collected and entered into the Paris Transplant Group database (French data protection authority registration number: 363505). The institutional review board of the Paris Institute for Transplantation and Organ Regeneration approved the study, and all patients gave written consent at the time of transplant. All data were pseudonymized and entered at the time of transplant by a senior nephrologist. Patients were followed up until May 1, 2021; death; or last medical contact.

#### Validation Cohorts

The external validation cohorts comprised 6363 individual who received kidney transplants from a living or deceased donor and were aged 18 years or older, representing all patients eligible for death risk evaluation from 8 centers in Europe and the US. French centers included Saint Louis and Bichat Hospitals, Paris (827 patients); Bretonneau Hospital, Tours (495 patients); and Toulouse Hospital, Toulouse (331 patients). European centers included Katholieke Universiteit Leuven, Belgium (1890 patients); Liege Hospital, Belgium (609 patients); and Leiden University Medical Center, Netherlands (1328 patients). US centers included the Hospital of the University of Pennsylvania, Philadelphia (383 patients), and Mayo Clinic, Phoenix, Arizona (500 patients). Patients included in validation cohorts underwent transplant between 2006 and 2023. Datasets for validation centers were collected from electronic health records, entered in center databases and sent anonymized to the Paris Transplant Group. In France, the transplant allocation system followed rules of the French National Agency for Organ Procurement.^13^ Details about external validation cohorts are presented in eMethods 3 in Supplement 1. Validation cohorts were obtained through established academic collaborations with participating centers. Each cohort was collected under local ethical approval at the respective centers in accordance with local regulations. Data were shared under data sharing and collaboration agreements between institutions. Individual patient consent was obtained as per requirements of each contributing center’s approved protocol.

#### Warehouse Validation Cohorts

We assembled 2 other validations cohorts comprising 4588 patients using the Greater Paris University Hospitals (GPUH) and University of California at San Francisco (UCSF) clinical data warehouses (CDWs). These CDWs are automatically filled databases containing data collected during routine clinical care at GPUH or UCSF medical centers. The UCSF cohort comprised 2028 US patients, and the GPUH cohort gathered 2560 patients who underwent transplants in 6 French transplant centers between 2017 and 2023. The list of codes used was adapted from previously published work^26,27^ and French National Health Insurance recommendations (eMethods 4 in Supplement 1). The UCSF warehouse validation cohort was obtained through established academic collaboration and collected under local ethical approval at the respective centers in acccordance with local regulations. Data were shared under data sharing and collaboration agreements between institutions. Individual patient consent was obtained as per requirements of each contributing center’s approved protocol. The GPUH CDW Scientific and Ethics Committee granted access to the CDW for the purpose of this study.

### Data Collected and Procedures

In the French derivation cohort, a total of 121 parameters were collected, including recipient or donor demographic characteristics, medical history, 37 biological parameters, and 2 imaging parameters. All recipient histories were collected manually in medical files based on the medical observation written on the day of admission and reviewed by senior nephrologists (C.D.D. and O.A.). Biological and imaging data were extracted from the electronic health record by trained research coordinators. All parameters included in this study were collected at or before the time of transplant. We measured all laboratory values using automated and standardized methods that are routinely performed as standard of care. Most laboratory values were measured at the day of transplant or, if not possible, during the week before transplant. No biological measurements were collected during or after the start of the surgery. The complete list of parameters is presented in eMethods 5 in Supplement 1.

In external validation cohorts, all baseline characteristics and parameters were collected as part of routine clinical care and recorded in local institutional databases in accordance with applicable local and national regulatory and ethical requirements. Collection was performed using electronic health records and completed manually. Datasets were subsequently fully anonymized and securely transferred to the Paris Transplant Group for centralized analysis. The GPUH CDW Scientific and Ethics Committee granted access to the CDW for this study.

### Outcome

The outcome of interest was kidney recipient death, occurring with or without functioning graft. Kidney recipient death was defined as all-cause mortality. In the derivation cohort, kidney recipient follow-up was defined as the time from the day of transplant until death, the end of follow-up, or May 1, 2021.

### Statistical Analysis

Continuous variables were described using means and SDs or medians and IQRs. We compared means and proportions between cohorts using Student *t* test, analysis of variance (Mann-Whitney test), or χ^2^ test (or Fisher exact test if appropriate). We used the Kaplan-Meier method to estimate patient survival. Statistical significance was set at *P* < .05, and all tests were 2 tailed. All analyses were performed using Stata statistical software version 17.0 (StataCorp) and R statistical software version 3.2.1 (R Project for Statistical Computing). Data were analyzed from January 2023 to June 2025.

In total, 20 591 of 241 164 data elements (8.5%) in the derivation cohort were missing. For patients with at least 1 missing data element for predictors of interest, the random survival forest imputation algorithm was performed.^28^ The number of trees was set to 500, and the maximum number of iterations was set to 10. There were no missing values regarding survival time or mortality status.

#### Development of a Patient Death Prediction Model

Cox regression analyses were performed to identify parameters associated with patient mortality. Parameters with *P* values less than .10 in univariable analysis were considered candidates for inclusion in multivariable analysis. The assumption of log linearity was tested for continuous variables (eMethods 6 in Supplement 1). The final variable selection was determined through stepwise backward selection from multivariable Cox regression, exploration of parameter combinations based on literature, and input from clinical and research experts (C.D.D., Ch.L., O.A., and A.L.). For highly correlated parameters, only one was retained based on correlation coefficients. Penalized regressions (least absolute shrinkage and selection operator [LASSO] and elastic net methods) were also performed to investigate the consistency and robustness of the selected variables (eMethods 7 in Supplement 1).

We then built the mortality box (mBox), a risk prediction model for kidney recipient death, using β regression coefficients estimated from the final multivariable Cox model (eMethods 8 in Supplement 1). Predicted probabilities were computed for each individual from 1 to 10 years after transplant. This time horizon was guided by the median (IQR) follow-up after transplant in the derivation cohort, which was 10.35 (7.67-13.53) years.

#### Internal and External Validation

The internal validity of the final multivariable model was confirmed by a bootstrap procedure using 1000 datasets resampled from the original dataset. We assessed the accuracy of the prediction model based on its discrimination ability using the Harrell concordance index (C statistic) and its calibration performance using visual examination of calibration plots (rms package in R statistical software) and calibration slopes. Brier scores were also calculated to assess the overall accuracy of predicted probabilities. Clinical utility was assessed using decision curves. Predictive performances were assessed from 1 to 10 years after transplant. For patients who were followed up for more than 10 years, follow-up was right censored at 10 years for performance evaluation.

The external validity of the final model was thereafter evaluated in external validation cohorts. To accommodate heterogeneity in data availability and clinical practices across external validation cohorts, abbreviated versions of the model were developed for centers where certain imaging or biological parameters were not routinely collected (eMethods 9 in Supplement 1).

#### Sensitivity Analyses

##### Performance in Additional Subpopulations

We investigated the performance of the model in several subpopulations. These were male and female patients, patients with an allograft from a living or deceased donor, patients with and without donor-specific antibodies, patients who had the outcome before and after the COVID-19 period, patients who underwent transplants before or after 2010, patients with and without hepatitis C virus (HCV) antibodies, and patients with a body mass index (BMI; calculated as weight in kilograms divided by height in meters squared) less than 25 or 25 or greater.

##### Comparison With Previously Published Mortality Scores and Other Modeling Approaches

Previously published mortality scores^15,16,17,21,23,29,30^ were applied to our derivation and validation cohorts to compare their performance with that of the study model (eMethods 10 in Supplement 1). We also investigated machine learning (ML) models adapted to time-to-event data to compare their predictive performances with that of the study model (eMethods 11 in Supplement 1).

## Results

### Characteristics of Derivation and Validation Cohorts

The study comprised 12 517 participants, including 1566 from the derivation cohort (mean [SD] age, 50.05 [14.31] years; 942 male [60.15%]) and  10 951 from the external validation cohorts (mean [SD] age, 53.32 [13.97] years; 6766 male [61.78%]). A total of 2486 patients (19.9%) died after a median (IQR) follow-up of 5.08 (2.97-7.00) years. Among 1566 donors, the mean (SD) age was 54.24 (17) years and 816 (52.11%) were male. A total of 346 kidney transplants (22.09%) were from living donors. The median (IQR) follow-up after transplant was 10.35 (7.67-13.53) years in the derivation cohort. External validation consisted of 1653 patients from 3 centers in France, 3827 patients from 3 centers in Europe, and 883 patients from 2 centers in the US. Additionally, 2 clinical data warehouses were included for external validation, including 2560 patients from 6 centers of the GPUH and 2028 patients of the UCSF. Baseline characteristics of derivation and validation cohorts are shown in Table 1 and eTables 1 to 5 in Supplement 1. Kaplan-Meier survival curves are shown in eFigure 1 in Supplement 1.

**Table 1.** Baseline Characteristics of Derivation and Validation Cohorts^a^

Characteristic	Derivation cohort (n = 1566)		Validation cohort						*P *value
			French (n = 1653)		European (n = 3827)		US (n = 883)		
	Total, No.	No. (%)	Total, No.	No. (%)	Total, No.	No. (%)	Total, No.	No. (%)	
Recipient demographics									
Age, mean (SD), y	1566	50.05 (14.31)	1653	52.95 (14.49)	3827	54.09 (13.19)	883	52.87 (14.05)	<.001
Sex									
Male	1566	942 (60.15)	1653	1064 (64.37)	3827	2415 (63.10)	883	487 (55.15)	<.001
Female		624 (39.85)		589 (35.63)		1412 (36.90)		396 (44.85)	
BMI, mean (SD)	1560	23.82 (4.44)	1640	24.96 (4.97)	3517	25.66 (4.46)	883	27.90 (5.76)	<.001
Dialysis	1537	1245 (81.00)	1653	1414 (85.54)	3825	3178 (83.08)	883	712 (80.63)	.002
Time since onset of dialysis, median (IQR), y	1534	3.19 (0.87-6.37)	1646	2.31 (0.85-4.24)	3825	2.08 (0.62-3.85)	881	1.75 (0.40-4.00)	<.001
End-stage kidney disease cause									
Glomerulonephritis	1566	440 (28.10)	1653	399 (24.14)	3218	1075 (33.41)	881	196 (22.25)	<.001
Diabetes		133 (8.49)		211 (12.76)		418 (12.99)		222 (25.20)	
CIN		255 (15.13)		113 (6.84)		236 (7.33)		18 (2.04)	
Vascular		69 (4.41)		158 (9.56)		148 (4.60)		136 (15.44)	
PKD		151 (9.64)		200 (12.10)		515 (16.00)		81 (9.19)	
Unknown		334 (21.33)		317 (19.18)		244 (7.58)		88 (9.99)	
Other		184 (11.75)		255 (15.43)		582 (15.2)		140 (15.44)	
Donor characteristics									
Age, mean (SD), y	1566	54.24 (17.00)	1653	54.05 (16.55)	3820	50.05 (14.21)	882	38.97 (15.64)	<.001
Sex									
Male	1566	816 (52.11)	1653	904 (54.69)	3816	2009 (52.65)	883	473 (53.57)	.44
Female		750 (47.89)		749 (45.31)		1807 (47.35)		410 (46.43)	
BMI, mean (SD)	1566	25.55 (4.99)	1651	25.74 (4.92)	3758	25.40 (4.05)	781	28.23 (7.64)	<.001
Living donor	1566	346 (22.09)	1653	290 (17.54)	3827	931 (24.33)	882	208 (23.58)	<.001
Transplant characteristics									
Transplant rank									
1	1566	1272 (81.23)	1653	1409 (85.24)	3827	3302 (86.28)	883	767 (86.86)	<.001
2		226 (14.43)		207 (12.52)		453 (11.84)		99 (11.21)	
3		60 (3.83)		34 (2.06)		61 (1.59)		14 (1.59)	
4		6 (0.38)		3 (0.18)		10 (0.26)		3 (0.34)	
5		2 (0.13)		0 (0)		1 (0.03)		0	
Anti-HLA donor-specific antibodies	1566	337 (21.52)	813	90 (11.07)	3786	235 (6.21)	883	76 (8.61)	<.001
Recipient history									
MACE PAD	1535	253 (16.48)	1653	387 (23.54)	3682	1081 (29.36)	883	185 (20.95)	<.001
Diabetes	1536	215 (14.00)	1649	375 (22.74)	3690	785 (21.27)	883	304 (34.43)	<.001
Atrial arrhythmia	1536	76 (4.95)	1649	158 (9.58)	1890	138 (7.30)	883	76 (8.61)	<.001
Cardiac valve disorder	1366	61 (4.47)	1647	180 (10.93)	2499	20 (0.80)	883	82 (9.29)	<.001
Psychiatric history	1535	89 (5.80)	1321	43 (3.26)	1890	332 (17.57)	882	120 (13.61)	<.001
HCV	1548	96 (6.20)	1645	67 (4.07)	2963	25 (0.84)	880	60 (6.82)	<.001
Biological variables									
Albumin, mean (SD), g/dL	1299	4.09 (0.58)	1459	4.18 (0.48)	3409	4.14 (0.56)	659	4.24 (0.53)	<.001
HbA_1c_, mean (SD), %	1170	5.63 (0.97)	1387	5.71 (1.05)	3643	5.54 (0.92)	427	5.80 (1.41)	<.001
Troponin positivity	1349	167 (12.38)	1156	170 (14.71)	1691	456 (26.97)	0	NA	<.001
CRP positivity	1309	275 (21.01)	1558	372 (23.88)	2220	652 (29.37)	0	NA	.002
GGT, median (IQR), U/L	1413	24 (16-38)	1588	23 (16-4.38)	3649	22 (15-35)	0	NA	<.001

Abbreviations: CIN, chronic interstitial nephropathy; CRP, C-reactive protein; GGT, γ-glutamyltransferase; HLA, human leukocyte antigen; MACE, major adverse cardiac event; PAD, peripheral artery disease; PKD, polycystic kidney disease.

SI conversion factors: To convert albumin to grams per liter, multiply by 10; GGT to microkatals per liter, multiply by 0.0167; HbA_1C_ to proportion of total hemoglobin, multiply by 0.01.

^a^ Characteristics of Greater Paris University Hospital and University of California at San Francisco database validation cohorts are in eTable 5 in Supplement 1. This table depicts recipient, donor, and transplant characteristics of the derivation cohort and external validation cohorts from France, Europe, and the US. Clinical data were collected from each center and entered into the Paris Transplant Group database. All data were anonymized.

In the derivation cohort, 504 patients died during the entire follow-up, and among these patients, the median (IQR) survival time until death was 5.89 (3.20-8.78) years. In validation cohorts, 1982 patients died and the median (IQR) follow-up time after transplant was 4.33 (1.97-7.49) years. At 5 years after transplant, 213 patients (13.6%) died in the derivation cohort vs 1214 patients (11.1%) in validation cohorts (190 patients [12.0%] in the French validation cohort, 442 patients [11.5%] in the European cohort, 84 patients [9.5%] in the US cohort, 343 patients [13.3%] in the GPUH cohort, and 147 patients [7.2%] in the UCSF cohort).

### Prediction of Mortality in Derivation Cohort

Of 121 candidate variables, 47 clinical and 21 biological variables were associated with mortality in univariable analysis (eTable 6 in Supplement 1) and 46 were selected for multivariable analysis. In the final multivariable model (Table 2), 14 variables were independently associated with patient death: recipient age (hazard ratio [HR] per 1-year increase in age, 1.07 [95% CI, 1.06-1.08]; *P* < .001); duration of dialysis prior to transplant (HR for dialysis >3 years vs no dialysis, 1.55 [95% CI, 1.13-2.11]; *P* = .006); history of major cardiovascular events, including stroke, myocardial infarction, and peripheral vascular disease (HR, 1.68 [95% CI, 1.36-2.09]; *P* < .001); history of atrial rhythm disorder, including atrial fibrillation or flutter, treated or not (HR, 1.47 [95% CI, 1.07-2.02]; *P* = .02); cardiac valvulopathy, including any cardiac valvular stenosis or insufficiency, mild or severe (HR, 1.51 [95% CI, 1.01-2.26]; *P* = .046); history of psychiatric disorder (HR, 2.23 [95% CI, 1.54-3.24]; *P* < .001); presence of anti-HCV antibodies (HR, 1.58 [95% CI, 1.13-2.21]; *P* = .008); value of left ventricular mass (HR per 1-g/m^2^ increase, 1.01 [95% CI, 1.003-1.01]; *P* < .001); kidney allograft length (HR per 0.1-m^3^/kg, 0.82 [95% CI, 0.72-0.93]; *P* = .002); and 5 biological variables: glycated hemoglobin level (HR per 1–percentage point increase, 1.21 [95% CI, 1.11-1.31]; *P* < .001), albumin level (HR per 1-g/L increase, 0.98 [95% CI, 0.96-0.99]; *P* = .01), positive troponin status (HR, 1.38 [95% CI, 1.06-1.80]; *P* = .02), positive C reactive protein status (HR, 1.28 [95% CI, 1.04-1.56]; *P* = .02), and γ-glutamyltransferase level (HR per 1–log unit increase, 1.39 [95% CI, 1.22-1.58]; *P* < .001). LASSO and elastic net approaches yielded a consistent set of predictors (eTables 7 and 8 in Supplement 1).

**Table 2.** Predictors of Patient Death Assessed at Time of Transplant in Derivation Cohort: Multivariable Analysis^a^

Predictor	No.		HR (95% CI)	*P* value	Internal validation, 95% CI
	Patients	Events^b^			
Recipient age at transplant, per 1-y increase	1566	414	1.07 (1.06-1.08)	<.001	1.06-1.08
Major adverse cardiovascular event	1566	414	1.68 (1.36-2.09)	<.001	1.34-2.10
Atrial rhythm disorder	1566	414	1.47 (1.07-2.02)	.02	1.05-1.99
Valvulopathy	1566	414	1.51 (1.01-2.26)	.04	0.95-2.27
Psychiatric disorder	1566	414	2.23 (1.54-3.24)	<.001	1.53-3.20
Positive HCV serology	1566	414	1.58 (1.13-2.21)	.008	1.09-2.25
Left ventricular mass, per 1-g/m^2^ increase	1566	414	1.01 (1.003-1.01)	<.001	1.003-1.008
Kidney transplant size/donor BMI, per 0.1-m^3^/kg increase	1566	414	0.82 (0.72-0.93)	.002	0.72-0.95
Dialysis time, y					
0	292	50	1 [Reference]	NA	NA
<3	450	123	1.18 (0.84-1.64)	.34	0.82-1.67
≥3	824	241	1.55 (1.13-2.11)	.006	1.13-2.16
HbA_1c_ level, per 1–percentage point increase	1566	414	1.21 (1.11-1.31)	<.001	1.11-1.32
Albumin level, per 1-g/dL increase	1566	414	0.98 (0.96-0.99)	.01	0.96-0.996
Troponin level, ng/mL					
≤0.05	1383	341	1 [Reference]	NA	NA
>0.05	183	73	1.38 (1.06-1.80)	.02	1.05-1.78
CRP level, mg/dL					
≤0.6	1054	234	1 [Reference]	NA	NA
>0.6	512	180	1.28 (1.04-1.56)	.02	1.02-1.59
GGT level, per log increase	1566	414	1.39 (1.22-1.58)	<.001	1.21-1.62

Abbreviations: BMI, body mass index (calculated as weight in kilograms divided by height in meters squared); CRP, C-reactive protein; HbA_1c_, hemoglobin A_1c_; HCV, hepatitis C virus; HR, hazard ratio; NA, not applicable.

SI conversion factors: To convert albumin to grams per liter, multiply by 10; HbA_1C_ to proportion of total hemoglobin, multiply by 0.01; troponin to micrograms per liter, multiply by 1.

^a^ This table shows parameters independently associated with patient death in the final model. The internal validity of the final multivariable model was confirmed by a bootstrap procedure using a generation of 1000 datasets resampled from the original dataset. Major cardiovascular events include major adverse cardiac and cerebrovascular events and peripheral arterial disease.

^b^ The No. of events at 10 years after transplant is given.

We calculated the model prognostic score for each patient according to β regression coefficients estimated from the final model. The distribution of the score is presented in eFigure 2 in Supplement 1.

### Internal Validation of the Model

**Figure 1.**
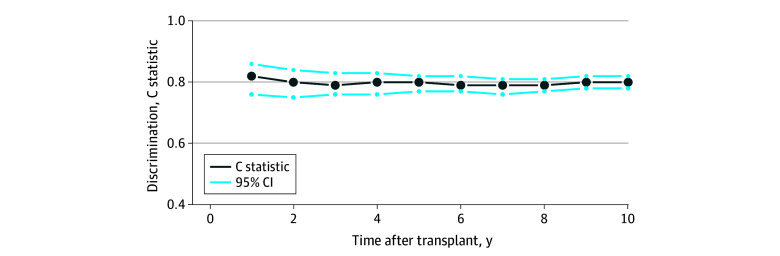
Line Graph of Model Discrimination Performance in Derivation Cohort This figure shows the discrimination of the model (that is, its ability to discriminate patients who are going to die from those who are not) from 1 to 10 years after transplant.

The final multivariable model was internally validated using a bootstrapping procedure with 1000 samples from the derivation cohort (Table 2). One variable, the presence of cardiac valvulopathy, had a CI that crossed 1 after bootstrap validation. Discrimination was assessed at each year after transplant up to 10 years, with a C statistic of 0.82 (95% CI, 0.77-0.87) at 1 year and 0.80 (95% CI, 0.78-0.82) at 10 years (Figure 1; eTable 9 in Supplement 1). Calibration showed good agreement between model-predicted probabilities and patient survival (eFigure 3, eTable 10 in Supplement 1). The Brier score over the 10-year follow-up was 0.13 (95% CI, 0.12-0.14) (eTable 10 in Supplement 1). Decision curve analysis demonstrated a positive net benefit throughout the range of threshold probabilities (eFigure 4 in Supplement 1).

### External Validation of the Model

To fit with routinely collected data in external validation cohorts, abbreviated models comprising 7 to 13 parameters were developed. The discrimination of abbreviated models in the derivation cohort ranged from C statistics of 0.78 (95% CI, 0.76-0.80) to 0.80 (95% CI, 0.77-0.81) compared with 0.80 (95% CI 0.78-0.82) for the main model, with good calibration (eTable 11 and eFigure 5 in Supplement 1) and comparable net benefit to that of the main model (eFigure 6 in Supplement 1).

**Figure 2.**
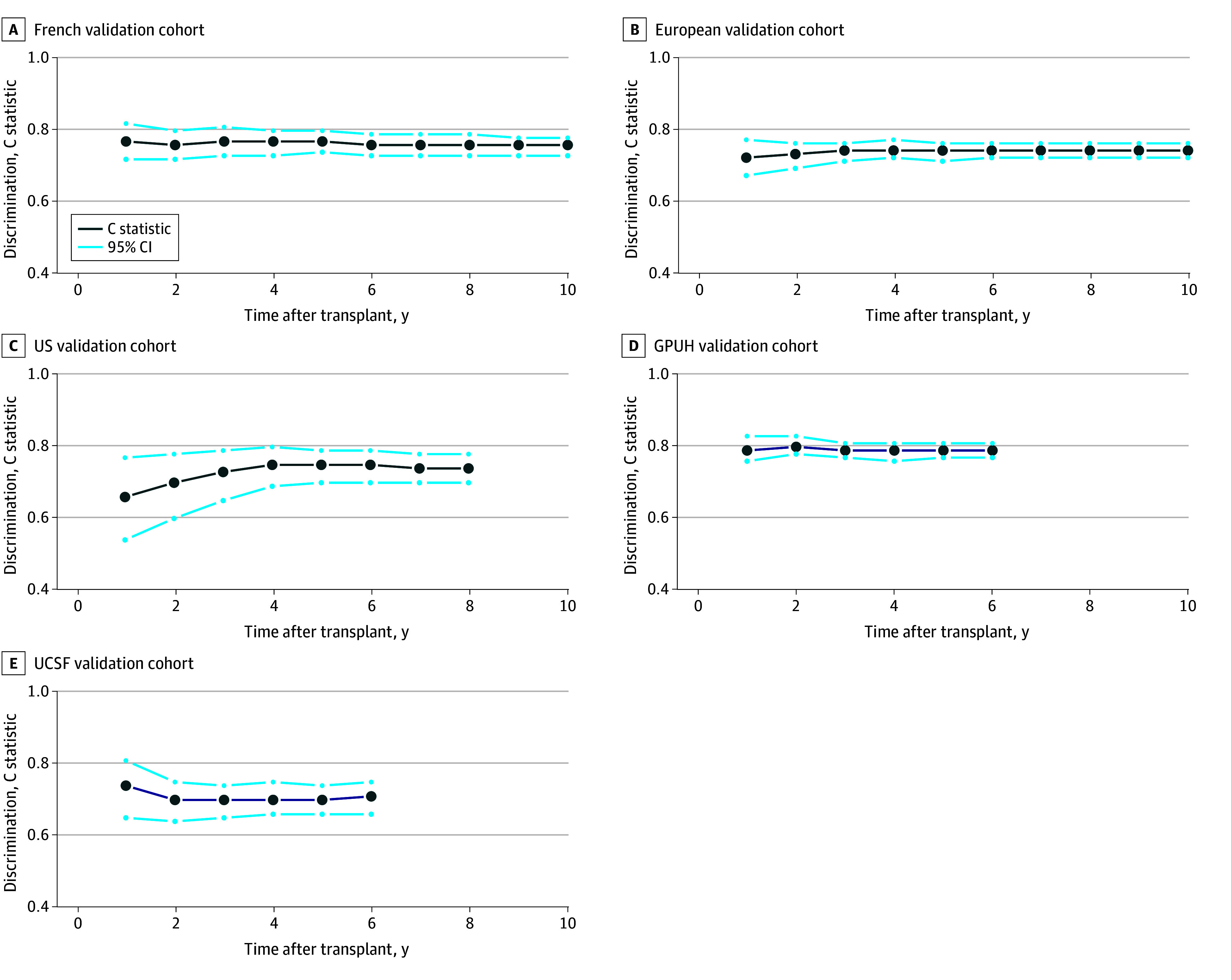
Line Graphs of Model Discrimination Performance in External Validation Cohorts This figure shows the discrimination of the model (that is, its ability to discriminate patients who are going to die from those who are not) in external validation cohorts, including France (A), Europe (B), North-America (C), the Greater Paris University Hospital (GPUH) database in France (D), and University of California at San Francisco (UCSF) database in the US (E).

The abbreviated models demonstrated consistent discrimination across all external validation cohorts (Figure 2; eTable 12 in Supplement 1). In the French validation cohort, C statistics were 0.77 (95% CI, 0.72-0.82) and 0.76 (95% CI, 0.73-0.78) at 1 year and 10 years after transplant, respectively. In the European cohort, C statistics were 0.72 (95% CI, 0.67-0.77) at 1 year and 0.74 (95% CI, 0.72-0.76) at 10 years. In the US cohort, C statistics were 0.66 (95% CI, 0.54-0.77) at 1 year and 0.74 (95% CI, 0.70-0.78) at 7 years. In clinical data warehouses, C statistics were 0.79 (95% CI, 0.76-0.83) at 1 year and 0.79 (95% CI, 0.77-0.81) at 3 years for GPUH and 0.74 (95% CI, 0.65-0.81) at 1 year and 0.70 (95% CI, 0.65-0.74) at 3 years for UCSF. Calibration curves are presented in eFigures 7 to 11 in Supplement 1. Brier scores were 0.15 (95% CI, 0.13-0.17) in French, 0.18 (95% CI, 0.17-0.19) in European, 0.13, (95% CI, 0.11-0.15) in US, 0.13 (95% CI, 0.11-0.14) in GPUH, and 0.11 (95% CI, 0.10-0.13) in UCSF validation cohorts (eTable 13 in Supplement 1).

Regarding clinical utility, the models maintained positive net benefit across threshold probabilities ranging from 0% to 50% in French, European, and GPUH warehouse validation cohorts. This ranged from 0% to 35% in the US validation cohort and from 0% to 22% in the UCSF warehouse validation cohort (eFigure 12 in Supplement 1).

### Sensitivity Analyses

#### Prediction Performances in Additional Clinical Scenarios and Subpopulations

Sensitivity analyses performed to test the robustness and generalizability of the model in different clinical scenarios and subpopulations are presented in Table 3. We tested the performance of the model in the following subgroups: living donor transplant (C statistic 0.81 [95% CI, 0.73-0.88]) and deceased donor transplant (C statistic, 0.78 [95% CI, 0.76-0.80]), patients with (C statistic, 0.78 [95% CI, 0.74-0.83]) or without (C statistic, 0.80 [95% CI, 0.78-0.83]) donor-specific antibodies at the time of transplant, women (C statistic, 0.81 [95% CI, 0.77-0.84]) and men (C statistics, 0.79 [95% CI, 0.77-0.82]), patients with a BMI of 25 or greater (C statistic, 0.77 [95% CI, 0.74-0.80]) and less than 25 (C statistic, 0.81 [95% CI, 0.78-0.83]), and patients who underwent transplants before (C statistic, 0.80 [95% CI, 0.77-0.83]) or after (C statistic, 0.79 [95% CI, 0.76-0.82]) 2010. We also assessed the performance of the model in predicting the outcome before (C statistic, 0.79; 95% CI, 0.77-0.81) and after (C statistic, 0.83 [95% CI, 0.77-0.90]) the pandemic, with March 2020 as the reference date. When recipient sex was added as a predictor in the model, no improvement in prediction performance was observed (eTables 14 and 15 and eFigures 13 and 14 in Supplement 1). Similarly, adding parameters assessed at the time of transplant, such as recipient initial nephropathy, was not associated with improved performance.

**Table 3.** Performances of the Model in Different Subpopulations and Clinical Scenarios^a^

Scenario or subpopulations	No.		C statistic (95% bootstrap percentile CI)
	Patients	Events	
Overall	1566	414	0.80 (0.78-0.82)
Living donor			
Yes	346	38	0.81 (0.73-0.88)
No	1220	376	0.78 (0.76-0.80)
Patient with DSA			
Yes	337	99	0.78 (0.74-0.83)
No	1229	315	0.80 (0.78-0.83)
Sex			
Women	624	156	0.81 (0.77-0.84)
Men	942	258	0.79 (0.77-0.82)
BMI			
≥25	542	180	0.77 (0.74-0.80)
<25	1021	234	0.81 (0.78-0.83)
No HCV	1566	414	0.80 (0.78-0.82)
Before COVID-19 era	1566	449	0.79 (0.77-0.81)
With unrestricted follow-up	1566	504	0.79 (0.77-0.81)
Transplant date			
Before 2010	910	217	0.80 (0.77-0.83)
On or after 2010	656	197	0.79 (0.76-0.82)

Abbreviations: BMI, body mass index (calculated as weight in kilograms divided by height in meters squared); DSA, donor-specific antibodies; HCV, hepatitis C virus.

^a^ This table shows the discrimination of the model (that is, its ability to discriminate patients who are going to die from those who are not) at 10 years after transplant in different clinical scenarios and subpopulations.

#### Previously Published Mortality Scores

Comparison of the model with previously developed mortality scores is presented in eTable 16 in Supplement 1. The 10-year discrimination ranged from 0.73 (95% CI, 0.70-0.75) for the score developed by Kasiske et al^15^ to 0.77 (95% CI, 0.75-0.79) for the Transplant Score^16^ compared with 0.80 (95% CI, 0.78-0.82) for the model.

#### Other Modeling Approaches

We also compared the model with ML approaches, including 6 ML models adapted for time-to-event data. These models showed similar discrimination and overall accuracy to the main model, with C statistics ranging from 0.77 (95% CI, 0.73-0.81) for random survival forest with extremely randomized trees to 0.78 (95% CI, 0.75-0.82) for extreme gradient boosting and Brier scores ranging from 0.14 (95% CI, 0.12-0.16) for random survival forest with extremely randomized trees to 0.15 (95% CI, 0.13-0.16) for random survival forest with maximally selected rank statistics across the ML models compared with 0.80 (95% CI, 0.78-0.82) and 0.13 (95% CI, 0.12-0.14), respectively, for our model (eTable 17 in Supplement 1). The most predictive parameters in the ML models, identified through variable importance, were consistent with independent predictors of mortality included in the main model (eFigure 15 in Supplement 1).

## Discussion

In this prognostic study, we developed a model for kidney transplant recipients that accurately predicted short- and long-term death after transplant and was computable on the day of transplant. The model demonstrated good prediction performance and was externally validated in 8 centers from France, Europe, and the US and in 2 cohorts with general population evidence and distinct recipient characteristics, allocation systems, and posttransplant treatment, demonstrating its robustness. The model was validated in many patient subpopulations and tested for diverse time frames.

Our model contrasts with previous kidney recipient death prediction models because of their significant shortcomings that hampered implementation in clinical practice. Prior models suffered from small patient cohorts,^20,23,24,31^ lack of validation in external cohorts and various subpopulations,^31,32^ short follow-up periods,^21,32^ and reliance on parameters not routinely assessed in clinical practice,^15,16,21,23,24,32^ which limited their generalizability. Furthermore, previous models exhibited limited prediction performance.^15,16,17,21,30,31,32,33^ This study was specifically designed to address these limitations.

Although recipient selection practices and kidney allocation policies vary among countries, they are often based on models that help match each donated organ with the best wait-listed candidate in an attempt to balance equitable distribution with optimal use.^34^ In France, the Kidney Score does not predict the risk of death after transplant.^35^ In the US, the Estimated Post-Transplant Survival (EPTS) model, which relies on age, prior transplant status, dialysis duration, and diabetes history, is used in combination with the Kidney Donor Profile Index to match kidney donors and recipients.^36^ However, the performance of the EPTS and Kidney Donor Profile Index is moderate to low (C statistics of 0.67 for EPTS alone and 0.69 for EPTS combined with KDRI^37^).

Consequently, because allocation policies may lack estimation of patient survival or may be based on inaccurate estimation of patient survival, our model could be associated with improved allocation efficiency because of its accuracy and validation in many patient populations and subgroups. Our model contains clinical, cardiovascular, biological, and imaging parameters that are independently associated with patient death. This shows that multiple distinct parameters may contribute to patient health decline and suggests that their integration in a single model is the best strategy to attain high prediction performance.

Importantly, some of these parameters, such as diabetes balance assessed through glycated hemoglobin or nutritional status assessed by the albumin level, may be modified while patients are on the waiting list. Therefore, we encourage adequate treatment of diabetes, optimal nutrition, and treatment of HCV infection before or rapidly after transplant to improve posttransplant survival, as suggested by the Kidney Disease: Improving Global Outcomes group.^38^

Of the 14 donor parameters tested, 12 were associated with recipient death in univariable models. However, only 1 remained associated in the final multivariable model, suggesting that the prediction of recipient death relies mostly on recipient characteristics.

This study has several strengths. First, we collected a large number of parameters; for each patient in the derivation cohort, all data were collected and manually verified in medical files by senior nephrologists. Moreover, as revealed in the literature search, we collected more information than any other study to our knowledge that developed a kidney recipient death–prediction model. Our model was externally validated in 4 countries, which is unprecedented, with prediction performance higher than that of any other published patient death prediction model to our knowledge. Furthermore, the model was designed to predict short- and long-term death at the time of transplant, which is also novel.

The model has potential for guiding posttransplant decisions due to its ability to provide a comprehensive risk profile of the kidney recipient at the time of transplant. The model could therefore assist clinicians in identifying patients who may benefit from closer monitoring and more personalized posttransplant care. For example, patients identified as higher risk by the model could be candidates for more frequent follow-ups or tailored clinical strategies aimed at addressing specific risks identified by the model. Ultimately, while the model is designed to predict outcomes at the time of transplant, its information could be useful in shaping subsequent care plans.

In addition, when considering how to use model predictions in clinical practice, a key question is whether and how to share risk probabilities with patients. While directly providing patients with these probabilities may promote transparency and empower informed decision-making, it also has the potential to cause unnecessary anxiety and self-limiting behavior. Conversely, withholding this information allows clinicians to use the data to guide care without burdening the patient with potential distress. However, this approach may limit patient autonomy. Therefore, a middle ground strategy may be preferred; this would involve patients in a discussion about how much information they wish to receive. By asking patients whether they want to know their specific risk probabilities, clinicians can respect individual preferences and empower patients to make decisions that align with their comfort level. This strategy allows for a personalized approach tailored to each patient’s desire for involvement in their care decisions. Because this issue is key, we will make sure to engage patient advocacy organizations on the subject of sharing our model’s information.

### Limitations

Several limitations should be acknowledged. First, some variables needed for the full model, such as troponin or hemoglobin A_1c_, may not be routinely available, which could hamper its deployment in some transplant centers. Therefore, to account for variability in clinical practice across transplant centers, we developed abbreviated models with good prediction performance, allowing computation in all external validation cohorts. Second, while the model was validated in 8 external validation centers in 4 other countries, it was not validated in Asia or South America, which should be investigated in the future. Third, although the presence of anti-HCV antibodies at the time of transplant was included in the model, HCV viremia data were not available. Fourth, the model was validated in cohorts with retrospectively collected data. A validation study in a prospective cohort with a systematic approach to data collection is needed to test the model’s performance in clinical conditions.

## Conclusions

In this prognostic study, we developed the mBox, a robust model predicting kidney transplant recipient death at the time of transplant. The model was validated in external validation cohorts from several countries and in many subpopulations and clinical scenarios. Assessing mortality risk at the time of transplant with this model may help clinicians better stratify patient risk of death and guide medical decisions.

## Notes

Supplement 1. eMethods 1. Literature review of mortality prediction models for kidney recipients eMethods 2. Study flowchart in the derivation cohort eMethods 3. External validation cohorts eMethods 4. List of diagnostics, procedures, and biology codes used to build the validation cohorts in clinical data warehouses eMethods 5. Candidate predictors eMethods 6. Management of biological variables for statistical analyses eMethods 7. Penalized regression methods eMethods 8. Construction of the integrative score from the multivariable Cox model eMethods 9. Abbreviated models eMethods 10. Previously published mortality prediction models eMethods 11. Machine learning models eTable 1. Baseline characteristics of the derivation cohort before and after missing data imputation eTable 2. Baseline characteristics of the French validation cohort eTable 3. Baseline characteristics of the European validation cohort eTable 4. Baseline characteristics of the US validation cohort eTable 5. Baseline characteristics of the clinical data warehouses validation cohorts eTable 6. Cox univariable analyses eTable 7. Selected variables with LASSO-penalized Cox model eTable 8. Selected variables with elastic net–penalized Cox model eTable 9. Time-dependent discrimination of the model in the derivation cohort eTable 10. Calibration and overall accuracy of the model in the derivation cohort (10-year prediction horizon) eTable 11. Performances of the abbreviated models in the derivation cohort (10-year prediction horizon) eTable 12. Time-dependent discrimination of the model in the external validation cohorts eTable 13. Calibration and overall accuracy of the model in the external validation cohorts (respectively 10-year, 7-year, and 5-year prediction horizon for France and Europe, US, and GPUH and UCSF) eTable 14. Multivariable model including recipient sex eTable 15. Performance of the model with and without recipient sex (10-year prediction horizon) eTable 16. Discrimination of previously published mortality-prediction models applied to derivation and validation cohorts eTable 17. Performance of machine learning models (10-year prediction horizon) eFigure 1. Kaplan-Meier survival curves in derivation and external validation cohorts eFigure 2. Distribution of the model score in the derivation cohort eFigure 3. Calibration of the model score in the derivation cohort from 1 to 10 years after transplant eFigure 4. Decision curve analysis of the model in the derivation cohort (10-year prediction horizon) eFigure 5. Calibration of the abbreviated models in the derivation cohort (10-year prediction horizon) eFigure 6. Decision curve analysis of the model and the abbreviated models in the derivation cohort (10-year prediction horizon) eFigure 7. Calibration of the model score in the French external validation cohort from 1 to 10 years after transplant eFigure 8. Calibration of the model score in the European external validation cohort from 1 to 10 years after transplant eFigure 9. Calibration of the model score in the US external validation cohort from 1 to 7 years after transplant eFigure 10. Calibration of the model score in the GPUH external validation cohort at 3 years after transplant eFigure 11. Calibration of the model score in the UCSF external validation cohort at 3 years after transplant eFigure 12. Decision curve analysis of the model in the derivation cohort (10-year prediction horizon) and in the external validation cohorts (respectively 10-year, 7-year, and 5-year prediction horizon for France and Europe, US, and GPUH and UCSF) eFigure 13. Calibration of the model and the model including recipient sex as a predictor in the derivation cohort (10-year prediction horizon) eFigure 14. Decision curve analysis of the model and the model including recipient sex as a predictor in the derivation cohort (10-year prediction horizon) eFigure 15. Variable importance of the machine learning models (top 10 most important variables)

Supplement 2.
Data Sharing Statement

## References

[1] Levin A, Tonelli M, Bonventre J, ; ISN Global Kidney Health Summit participants. Global kidney health 2017 and beyond: a roadmap for closing gaps in care, research, and policy. Lancet. 2017;390(10105):1888-1917. doi:10.1016/S0140-6736(17)30788-2 28434650

[2] Heldal K, Hartmann A, Grootendorst DC, . Benefit of kidney transplantation beyond 70 years of age. Nephrol Dial Transplant. 2010;25(5):1680-1687. doi:10.1093/ndt/gfp681 20038521 PMC2856560

[3] Ojo AOHJ, Hanson JA, Wolfe RA, Leichtman AB, Agodoa LY, Port FK. Long-term survival in renal transplant recipients with graft function. Kidney Int. 2000;57(1):307-313. doi:10.1046/j.1523-1755.2000.00816.x 10620213

[4] Port FKWR, Wolfe RA, Mauger EA, Berling DP, Jiang K. Comparison of survival probabilities for dialysis patients vs cadaveric renal transplant recipients. JAMA. 1993;270(11):1339-1343. doi:10.1001/jama.1993.03510110079036 8360969

[5] Wolfe RAAV, Ashby VB, Milford EL, . Comparison of mortality in all patients on dialysis, patients on dialysis awaiting transplantation, and recipients of a first cadaveric transplant. N Engl J Med. 1999;341(23):1725-1730. doi:10.1056/NEJM199912023412303 10580071

[6] Laupacis A, Keown P, Pus N, . A study of the quality of life and cost-utility of renal transplantation. Kidney Int. 1996;50(1):235-242. doi:10.1038/ki.1996.307 8807593

[7] Evans RWMD, Manninen DL, Garrison LP Jr, . The quality of life of patients with end-stage renal disease. N Engl J Med. 1985;312(9):553-559. doi:10.1056/NEJM198502283120905 3918267

[8] Haller M, Gutjahr G, Kramar R, Harnoncourt F, Oberbauer R. Cost-effectiveness analysis of renal replacement therapy in Austria. Nephrol Dial Transplant. 2011;26(9):2988-2995. doi:10.1093/ndt/gfq780 21310740

[9] Cour des Comptes. L’insuffisance rénale chronique terminale: une prise en charge à réformer au bénéfice des patients. In: Rapport Public Annuel 2020 de la Cour des Comptes. Cour des Comptes; 2020:96-129. Accessed April 3, 2026. https://www.ccomptes.fr/system/files/2020-02/20200225-03-TomeI-insuffisance-renale-chronique-terminale.pdf

[10] Sørensen VR, Heaf J, Wehberg S, Sørensen SS. Survival benefit in renal transplantation despite high comorbidity. Transplantation. 2016;100(10):2160-2167. doi:10.1097/TP.0000000000001002 26599492 PMC5120769

[11] Tonelli M, Wiebe N, Knoll G, . Systematic review: kidney transplantation compared with dialysis in clinically relevant outcomes. Am J Transplant. 2011;11(10):2093-2109. doi:10.1111/j.1600-6143.2011.03686.x 21883901

[12] Lentine KL, Smith JM, Miller JM, . OPTN/SRTR 2021 annual data report: kidney. Am J Transplant. 2023;23(2)(suppl 1):S21-S120. doi:10.1016/j.ajt.2023.02.004 37132350 PMC9970360

[13] Agence de la Biomédecine. Le rapport médical et scientifique 2022 est en ligne. 2023. Accessed March 24, 2026. https://www.agence-biomedecine.fr/fr/institutionnel/le-rapport-medical-et-scientifique-2022-est-en-ligne

[14] Machnicki G, Pinsky B, Takemoto S, . Predictive ability of pretransplant comorbidities to predict long-term graft loss and death. Am J Transplant. 2009;9(3):494-505. doi:10.1111/j.1600-6143.2008.02486.x 19120083

[15] Kasiske BLIA, Israni AK, Snyder JJ, Skeans MA, Peng Y, Weinhandl ED. A simple tool to predict outcomes after kidney transplant. Am J Kidney Dis. 2010;56(5):947-960. doi:10.1053/j.ajkd.2010.06.020 20801565

[16] Molnar MZND, Nguyen DV, Chen Y, . Predictive score for posttransplantation outcomes. Transplantation. 2017;101(6):1353-1364. doi:10.1097/TP.0000000000001326 27391198 PMC5219861

[17] Baskin-Bey ESKW, Kremers W, Nyberg SL. A recipient risk score for deceased donor renal allocation. Am J Kidney Dis. 2007;49(2):284-293. doi:10.1053/j.ajkd.2006.10.018 17261431

[18] Gu H, Akhtar M, Shah A, Mallick A, Ostermann M, Chambers J. Echocardiography predicts major adverse cardiovascular events after renal transplantation. Nephron Clin Pract. 2014;126(1):75-80. doi:10.1159/000358885 24642876

[19] Malyala R, Rapi L, Nash MM, Prasad GVR. Pre-transplant left ventricular geometry and major adverse cardiovascular events after kidney transplantation. Ann Transplant. 2019;24:100-107. doi:10.12659/AOT.913649 30787265 PMC6397615

[20] Laging M, Kal-van Gestel JA, van de Wetering J, . A high comorbidity score should not be a contraindication for kidney transplantation. Transplantation. 2016;100(2):400-406. doi:10.1097/TP.0000000000000973 26516673

[21] Patzer RE, Basu M, Larsen CP, . iChoose Kidney: a clinical decision aid for kidney transplantation versus dialysis treatment. Transplantation. 2016;100(3):630-639. doi:10.1097/TP.0000000000001019 26714121 PMC4658512

[22] Pieloch D, Dombrovskiy V, Osband AJ, . The Kidney Transplant Morbidity Index (KTMI): a simple prognostic tool to help determine outcome risk in kidney transplant candidates. Prog Transplant. 2015;25(1):70-76. doi:10.7182/pit2015462 25758804

[23] Bamoulid J, Frimat M, Courivaud C, . A simple score to predict early death after kidney transplantation. Eur J Clin Invest. 2020;50(11):e13312. doi:10.1111/eci.13312 32533894

[24] Schwager Y, Littbarski SA, Nolte A, . Prediction of three-year mortality after deceased donor kidney transplantation in adults with pre-transplant donor and recipient variables. Ann Transplant. 2019;24:273-290. doi:10.12659/AOT.913217 31097680 PMC6540619

[25] Longenecker JC, Coresh J, Klag MJ, . Validation of comorbid conditions on the end-stage renal disease medical evidence report: the CHOICE study: Choices for Healthy Outcomes in Caring for ESRD. J Am Soc Nephrol. 2000;11(3):520-529. doi:10.1681/ASN.V113520 10703676

[26] Bannay A, Chaignot C, Blotière PO, . The best use of the Charlson Comorbidity Index with electronic health care database to predict mortality. Med Care. 2016;54(2):188-194. doi:10.1097/MLR.0000000000000471 26683778

[27] Harper C, Mafham M, Herrington W, . Comparison of the accuracy and completeness of records of serious vascular events in routinely collected data vs clinical trial-adjudicated direct follow-up data in the UK: secondary analysis of the ASCEND randomized clinical trial. JAMA Netw Open. 2021;4(12):e2139748. doi:10.1001/jamanetworkopen.2021.39748 34962561 PMC8715347

[28] Stekhoven DJ, Bühlmann P. MissForest—non-parametric missing value imputation for mixed-type data. Bioinformatics. 2012;28(1):112-118. doi:10.1093/bioinformatics/btr597 22039212

[29] Charlson ME, Pompei P, Ales KL, MacKenzie CR. A new method of classifying prognostic comorbidity in longitudinal studies: development and validation. J Chronic Dis. 1987;40(5):373-383. doi:10.1016/0021-9681(87)90171-8 3558716

[30] Bae S, Massie AB, Thomas AG, . Who can tolerate a marginal kidney: predicting survival after deceased donor kidney transplant by donor-recipient combination. Am J Transplant. 2019;19(2):425-433. doi:10.1111/ajt.14978 29935051 PMC6309666

[31] Haller MC, Wallisch C, Mjøen G, . Predicting donor, recipient and graft survival in living donor kidney transplantation to inform pretransplant counselling: the donor and recipient linked iPREDICTLIVING tool—a retrospective study. Transpl Int. 2020;33(7):729-739. doi:10.1111/tri.13580 31970822 PMC7383676

[32] Bui K, Kilambi V, Rodrigue JR, Mehrotra S. Patient functional status at transplant and its impact on posttransplant survival of adult deceased-donor kidney recipients. Transplantation. 2019;103(5):1051-1063. doi:10.1097/TP.0000000000002397 30086093 PMC6363903

[33] Koo TY, Lee J, Yang J. Development of predictive score for post-transplant survival based on pre-transplant recipient characteristics. Korean J Transplant. 2021;35(2):86-92. doi:10.4285/kjt.21.0011 35769528 PMC9235339

[34] Asfour NW, Zhang KC, Lu J, . Association of race and ethnicity with high longevity deceased donor kidney transplantation under the US kidney allocation system. Am J Kidney Dis. 2024;84(4):416-426. doi:10.1053/j.ajkd.2024.02.017 38636649 PMC11421570

[35] Agence de la Biomédecine. Guide du score rein 2020. Accessed March 12, 2026. https://www.agence-biomedecine.fr/IMG/pdf/guide_score_rein_v1.pdf

[36] Health Resources and Services Administration. KDPI calculator. Accessed March 24, 2026. https://hrsa.unos.org/data/allocation-calculators/kdpi-calculator/

[37] Clayton PA, McDonald SP, Snyder JJ, Salkowski N, Chadban SJ. External validation of the estimated posttransplant survival score for allocation of deceased donor kidneys in the United States. Am J Transplant. 2014;14(8):1922-1926. doi:10.1111/ajt.12761 24903739

[38] Awan AAY, Berenguer MC, Bruchfeld A, . Prevention, diagnosis, evaluation, and treatment of hepatitis C in chronic kidney disease: synopsis of the Kidney Disease: Improving Global Outcomes 2022 Clinical Practice Guideline. Ann Intern Med. 2023;176(12):1648-1655. doi:10.7326/M23-2391 38079642

