# Supplementary material for: A Prediction Model for Risk of Death in Kidney Transplant Recipients
Source: JAMA Netw Open. 2026 Apr 23;9(4):e267452. doi: 10.1001/jamanetworkopen.2026.7452 (PMC13107225; doi:10.1001/jamanetworkopen.2026.7452)
Supplement: Supplement 1. — eMethods 1. Literature review of mortality prediction models for kidney recipients eMethods 2. Study flowchart in the derivation cohort eMethods 3. External validation cohorts eMethods 4. List of diagnostics, procedures, and biology codes used to build the validation cohorts in clinical data warehouses eMethods 5. Candidate predictors eMethods 6. Management of biological variables for statistical analyses eMethods 7. Penalized regression methods eMethods 8. Construction of the integrative score from the multivariable Cox model eMethods 9. Abbreviated models eMethods 10. Previously published mortality prediction models eMethods 11. Machine learning models eTable 1. Baseline characteristics of the derivation cohort before and after missing data imputation eTable 2. Baseline characteristics of the French validation cohort eTable 3. Baseline characteristics of the European validation cohort eTable 4. Baseline characteristics of the US validation cohort eTable 5. Baseline characteristics of the clinical data warehouses validation cohorts eTable 6. Cox univariable analyses eTable 7. Selected variables with LASSO-penalized Cox model eTable 8. Selected variables with elastic net–penalized Cox model eTable 9. Time-dependent discrimination of the model in the derivation cohort eTable 10. Calibration and overall accuracy of the model in the derivation cohort (10-year prediction horizon) eTable 11. Performances of the abbreviated models in the derivation cohort (10-year prediction horizon) eTable 12. Time-dependent discrimination of the model in the external validation cohorts eTable 13. Calibration and overall accuracy of the model in the external validation cohorts (respectively 10-year, 7-year, and 5-year prediction horizon for France and Europe, US, and GPUH and UCSF) eTable 14. Multivariable model including recipient sex eTable 15. Performance of the model with and without recipient sex (10-year prediction horizon) eTable 16. Discrimination of previously published mortalit [file jamanetwopen-e267452-s001.pdf]

Supplemental Online Content

Debiais-Deschamps C, Raynaud M, Truchot A, et al. A prediction model for risk of death in kidney transplant recipients. *JAMA Netw Open*. 2026;9(4):e267452. doi:10.1001/jamanetworkopen.2026.7452

eMethods 1. Literature review of mortality prediction models for kidney recipients
eMethods 2. Study flowchart in the derivation cohort
eMethods 3. External validation cohorts
eMethods 4. List of diagnostics, procedures, and biology codes used to build the validation cohorts in clinical data warehouses
eMethods 5. Candidate predictors
eMethods 6. Management of biological variables for statistical analyses
eMethods 7. Penalized regression methods
eMethods 8. Construction of the integrative score from the multivariable Cox model
eMethods 9. Abbreviated models
eMethods 10. Previously published mortality prediction models
eMethods 11. Machine learning models
eTable 1. Baseline characteristics of the derivation cohort before and after missing data imputation
eTable 2. Baseline characteristics of the French validation cohort
eTable 3. Baseline characteristics of the European validation cohort
eTable 4. Baseline characteristics of the US validation cohort
eTable 5. Baseline characteristics of the clinical data warehouses validation cohorts
eTable 6. Cox univariable analyses
eTable 7. Selected variables with LASSO-penalized Cox model
eTable 8. Selected variables with elastic net–penalized Cox model
eTable 9. Time-dependent discrimination of the model in the derivation cohort
eTable 10. Calibration and overall accuracy of the model in the derivation cohort (10-year prediction horizon)
eTable 11. Performances of the abbreviated models in the derivation cohort (10-year prediction horizon)
eTable 12. Time-dependent discrimination of the model in the external validation cohorts
eTable 13. Calibration and overall accuracy of the model in the external validation cohorts (respectively 10-year, 7-year, and 5-year prediction horizon for France and Europe, US, and GPUH and UCSF)
eTable 14. Multivariable model including recipient sex
eTable 15. Performance of the model with and without recipient sex (10-year prediction horizon)
eTable 16. Discrimination of previously published mortality-prediction models applied to derivation and validation cohorts

© 2026 Debiais-Deschamps C et al. *JAMA Network Open*.

eTable 17. Performance of machine learning models (10-year prediction horizon)
eFigure 1. Kaplan-Meier survival curves in derivation and external validation cohorts
eFigure 2. Distribution of the model score in the derivation cohort
eFigure 3. Calibration of the model score in the derivation cohort from 1 to 10 years after transplant
eFigure 4. Decision curve analysis of the model in the derivation cohort (10-year prediction horizon)
eFigure 5. Calibration of the abbreviated models in the derivation cohort (10-year prediction horizon)
eFigure 6. Decision curve analysis of the model and the abbreviated models in the derivation cohort (10-year prediction horizon)
eFigure 7. Calibration of the model score in the French external validation cohort from 1 to 10 years after transplant
eFigure 8. Calibration of the model score in the European external validation cohort from 1 to 10 years after transplant
eFigure 9. Calibration of the model score in the US external validation cohort from 1 to 7 years after transplant
eFigure 10. Calibration of the model score in the GPUH external validation cohort at 3 years after transplant
eFigure 11. Calibration of the model score in the UCSF external validation cohort at 3 years after transplant
eFigure 12. Decision curve analysis of the model in the derivation cohort (10-year prediction horizon) and in the external validation cohorts (respectively 10-year, 7-year, and 5-year prediction horizon for France and Europe, US, and GPUH and UCSF)
eFigure 13. Calibration of the model and the model including recipient sex as a predictor in the derivation cohort (10-year prediction horizon)
eFigure 14. Decision curve analysis of the model and the model including recipient sex as a predictor in the derivation cohort (10-year prediction horizon)
eFigure 15. Variable importance of the machine learning models (top 10 most important variables)

This supplemental material has been provided by the authors to give readers additional information about their work.

eMethods 1. Literature review of mortality prediction models for kidney recipients

The table presents the literature review of existing mortality prediction scores computable at the time of transplantation.

© 2026 Debiais-Deschamps C et al. *JAMA Network Open*.

Study	Baskin-Bey et al. 2007	Kasiske et al. 2010	Pieloch et al 2015	Laging et al. 2015	Patzer et al. 2016	Schwager et al. 2019	Molnar et al. 2017	Bae et al. 2019	Bui et al. 2019	Ducloux et al. 2020	Haller et al. 2020	Koo et al. 2021	Miller et al. 2023
Name	RRS	-	KTMI	RoCKeT score	iChoose Kidney	-	Transplant score	EPTS & KDPI	-	ORLY score	Ipredictliving tool	K-EPTS	KTOP
Country	US	US	US	Netherlands	US	Germany	US	US	US	France	Norway	Corea	EU including Austria, Belgium, Germany
Time of computation	Day 0	Day 0 (or day 7 or 1 year)	Day 0	Day 0	Day 0	Day 0	Day 0	Day 0	Day 0	Day 0	Day 0	Day 0	Day 0
Transplantation period	1995-2002	2000-2006	2000 – 2008	2000-2013	2005-2011	2000-2012	2001-2006	2005-2016	2007-2014		1980--2007	2010-2018	2010-2018
Numbers of patients	47,535	59,091	100,261	1,728	57,479	1,546	15,215	120,818	72,839<br>53,242	942	837	6,731	32 958
Primary outcome	Patient and graft survival	Graft loss or death with functioning graft during the first 5 years	Graft loss and 3-years mortality	Mortality	1 and 3-years mortality Compared with dialysis	3-years mortality	5-years mortality Graft loss	5 and 10-years mortality	1 and 3 years mortality	Death at 3 years	Death (only living donation)	Mortality	Mortality
Recipient parameters	Age<br>Dialysis duration<br>Diabetes mellitus	Age<br>Dialysis duration<br>Cause of CKD	Age<br>Dialysis duration<br>BMI<br>Diabetes	9 comorbidities 3 associated:	Age<br>Ethnicity<br>Sex<br>Dialysis	Age<br>Diabetic Nephropathy<br>High immunisatio	Age<br>Ethnicity<br>Cause and duration of CKD	EPTS<br>Age<br>Diabete<br>Dialysis duration	KPSI<br>SRTR model (47 covariates for 1-year	Age<br>Sex<br>Diabetes<br>Dialysis vintage	Age<br>Sex<br>Year of of transplantation	Age<br>Diabetes<br>HCV<br>Dialysis vintage	Age<br>Sex<br>Diabetes<br>Cystic disease

© 2026 Debiais-Deschamps C et al. *JAMA Network Open*.

	History of Angina	(including diabetes)<br>HCV antibodies<br>Hypertension<br>Insurance<br>Ethnicity	PAD<br>Stroke<br>Coronaropathy<br>Previous Transplantation<br>functional Status	Cardiovascular disease<br>Other organ transplantation<br>Total of comorbidities	Comorbidities (diabetes, Hypertension, Cardiovascular disease)<br>Albumin <3,5g/dL	n<br>(TGI>85%)<br>Emergency	Dialysis duration<br>Diabetes<br>Coronary Artery Disease<br>PAD<br>Albumin<br>Insurance	Prior transplantation<br>(4 variables)	outcome or 61 covariates for 3-year outcome, from donor and recipient)	Chronic respiratory failure	Dialysis vintage<br>Cause of CKD<br>PAD<br>Cerebrovascular disease		Re transplantation
**Donor parameters**	Combined with Deceased Donor Score (5 variables : Age, Hypertension, last creatinine, cause of death and HLA mismatches)	Age<br>Hypertension<br>Trauma as cause<br>Of death	-	-	-	-	Age<br>Diabetes<br>ECD	KDPI (10 variables)	-	-	-	-	Age
**Transplant parameters**	-	HLA mismatches number	-	-	-	1 or 2 HLA DR mismatches<br>Cold ischemia time	HLA mismatches number	-	-	-	HLA mismatches	-	-
**Model**	Cox	Cox	Kaplan-Meier<br>Cox	Kaplan-Meier<br>Cox	Logistic Regression	Cox	Cox	Random Forest	Cox	Fine & Gray model	Cox	Cox	Cox

© 2026 Debiais-Deschamps C et al. *JAMA Network Open*.

**Internal validation**	-	Cross validation	-	-	-	-	Cross validation	-	-	Bootstrap	Bootstrap	-	-
**Missing value handling**	-	Mean imputation	-	-	-	-	Multiple imputation	Multiple imputation	Multiple imputation	-	Multiple imputation	-	-
**Discrimination**	C-stat = 0.692	C-stat = 0.649	-	-	C-stat=0.70	AUC : 0.75	C-stat = 0.70	C-stat = 0.637	Cstat = 0.710 (1year) 0.707 (3years)	C-stat = 0.78 (0.75-0.81)	C-stat = 0.77	C-stat = 0.69 (0.67-0.72)	AUC = 0.73-0.75 at 5 years 0.78-0.81 at 10-years
**Calibration**	No	Yes	No	No	No	No	Yes	No	Yes	Yes	No	No	No

BMI: Body Mass Index; EU: European Union; HCV: Hepatitis C Virus; KPSI: Karnovski Performance Score Index; PAD: Peripheral Arterial Disease

© 2026 Debiais-Deschamps C et al. *JAMA Network Open*.

The table presents the parameters used in existing mortality prediction scores computable at the time of transplantation.

	Baskin-Bey et al. RRS	Kasiske et al.	Pieloch et al. KTMI	Laging et al. RoCKeT score	KDRI	EPTS	Patzer et al. iChoose Kidney	Schwager et al.	Molnar et al. Transplant score	Bui et al.	Ducloux et al. ORLY	Haller et al. i-Predict Living Tool	Koo et al. K-EPTS	Miller et al. KTOP
**Donor parameters**														
**Age**		X			X				X	X				X
**Sex**										X				
**Ethnicity**					X					X				
**Height**					X					X				
**Weight**					X					X				
**Hypertension**		X			X					X				
**Diabetes**					X				X	X				
**HCV status**					X					X				
**Cardiac death**					X					X				
**Cause of death**		X			X					X				
**ECD**									X					
**Creatinin**					X			X		X				
**Blood type**										X				
**Recipient parameters**														
**Age**	X	X	X			X	X	X	X	X	X	X	X	X
**Sex**							X				X	X		X
**BMI**			X											
**Ethnicity**		X					X		X	X				
**HCV status**		X											X	
**HIV status**				x						X				
**Dialysis duration**	X	X	X			X	X		X		X	X	X	
**ESRD time**										X				

© 2026 Debiais-Deschamps C et al. *JAMA Network Open*.

**Initial nephropathy**		X							X	X		X		X
**Previous transplantation**			X	X		X				X				X
**Diabetes**	X		X	X		X	X	X	X	X	X		X	
**Hypertension**		X					X							
**Cardiovascular disease**							X							
**Coronaropathy**	X		X	X					X					
**Stroke**			X	X								X		
**Peripheral Arterial Disease**			X	X					X	X		X		
**Dependancy level**			X											
**KPSI**														
**Insurance**		X							X	X				
**CPRA**								X		X				
**Urgent transplantation**								X						
**Hepatic comorbidity**				X										
**Pulmonary comorbidity**				X							X			
**Cancer**				X										
**Transplant parameters**														
**Year of transplantation**												X		
**HLA mismatches**			X	X				X	X					

© 2026 Debiais-Deschamps C et al. *JAMA Network Open*.

**Cold ischemia time**			X					X		X				
**Biological parameters**														
**Albumin**							X		X	X				

BMI: Body Mass Index; CPRA: Calculated Panel Reactive Antibody; ECD: Extended Criteria Donor; ESRD: End Stage Renal Disease; HCV: Hepatitis C Virus; HIV: Human Immunodeficiency Virus; HLA: Human Leukocyte Antigens; KPSI: Karnovski Performance Score Index

© 2026 Debiais-Deschamps C et al. *JAMA Network Open*.

eMethods 2. Study flow chart in the derivation cohort

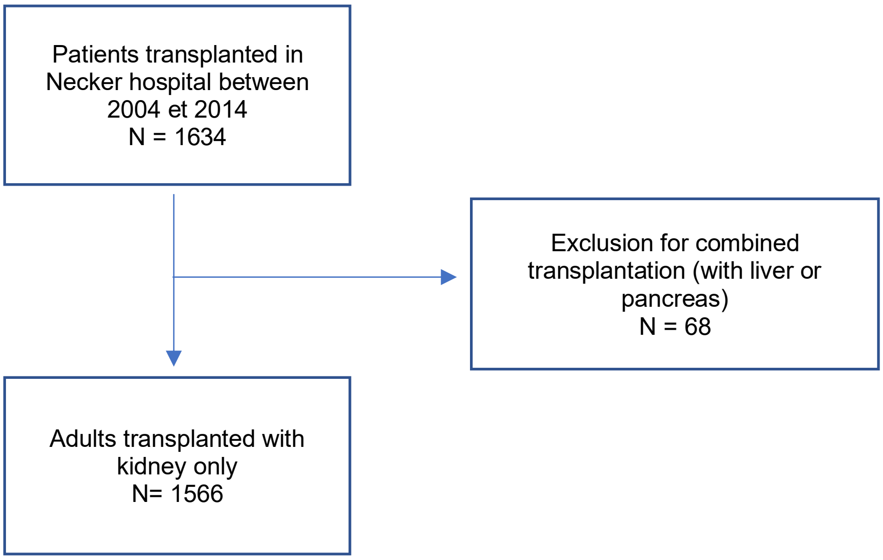

eMethods 3. External validation cohorts

The table depicts the characteristics of the external validation cohorts.

Country	Centre	Transplantation period	Number of patients	Follow up time in years, median (IQR)
France	Saint-Louis and Bichat Hospitals	2007-2014	827	8.5 (6.4-11.0)
	Tours Hospital	2014-2017	495	5.8 (4.9-6.9)
	Toulouse Hospital	2017-2022	331	1.7 (1.1-2.6)
Europe	Leuven Hospital (Belgium)	2006-2021	1,890	6.4 (3.4-10.8)
	Liege Hospital (Belgium)	2012-2022	609	8.4 (5.5-11.3)
	Leiden Hospital (Netherlands)	2012-2022	1,328	5.0 (2.0-7.0)
US	Hospital of the University of Pennsylvania (Philadelphia)	2015-2017	383	6.7 (6.2-7.4)
	Mayo Clinic (Phoenix)	2016-2017	500	6.0 (5.4-6.6)
Warehouses	Greater Paris University Hospitals (GPUH)	2017-2021	2,560	2.9 (1.6-4.1)
	University of California at San Francisco (UCSF)	2017-2023	2,028	2.7 (1.3-4.1)

© 2026 Debiais-Deschamps C et al. *JAMA Network Open*.

eMethods 4. List of diagnostics, procedures and biology codes used to build the validation cohorts in the clinical data warehouses

Measurement (Nomenclature)	Targeting	Input, before/at the time of transplantation
**Biology (LOINC)**		**HCV serology**: 13955-0, 16128-1, 16129-9, 16936-7, 22327-1, 40726-2, 5198-7, 72376-7<br>**HbA1c**: 17856-6, 4548-4, 4549-2, 62388-4<br>**Albumin**: 101198-0, 1751-7, 2862-1, 61151-7, 61152-5, 76631-1, 77148-5<br>**CRP**: 1988-5, 48421-2<br>**Troponin**: 10839-9, 42757-5, 67151-1<br>**Gamma-GT**: 2324-2
**Procedures (CPT [UCSF])**	**Kidney transplantation**: 50360, 50365<br>**Other SOT**: 47135, 44135, 44136, 48554, 32851, 32852, 32853, 32854, 33945	**Time on dialysis**: 90935, 90937, 90940, 90945, 90947, 90989, 90993, 90999<br>**Major adverse cardiovascular event (MACE)**: as detailed in CMS.gov A56823, A57590, A57180
**Procedures (CCAM [GPUH])**	**Kidney transplantation**: JAEA003<br>**Other SOT**: HLEA, HGEA, HNEA, HNEH, ZZEA, DZEA, GFEA	**Time on dialysis**: JVJF004, JVJF008, JVRP004, JVRP007, JVRP008, JVJB001<br>**Major adverse cardiovascular event (MACE)**: DDMA, DDAF, DDQH006, DDQH011, DDQH013, DDQH014, DDQH015, EEAF, EEPF, EEFA, EEJF, EENF, EECA, EDAA, EDEA, DGFA, EDFA, EDPF, EANF, EBAF, EBFA, EBCA<br>**Rhythm disorder**: DEPF
**Diagnoses (ICD-10)**		**MACE**: I22, I23, I24, I25, I63, I64, G450, G451, G452, G453, G458, G459, I702, I739, I740, I743, I744, I745<br>**Psychiatric disorder**: F10, F120, F15, F17, F00, F01, F02, F03, F05<br>**Valvular disorder**: I05, I06, I07, I08, I34, I35, I36, I37, I38, I39<br>**Rythm disorder**: I470, I471, I48<br>**Time on dialysis**: Z992
**Outcome**	Death (in or out of hospital)	

GPUH: Greater Paris University Hospitals; UCSF: University of California at San Francisco

© 2026 Debiais-Deschamps C et al. *JAMA Network Open*.

eMethods 5. Candidate predictors

The table presents the parameters collected in the derivation cohort. Atrial rhythm disorder includes atrial fibrillation or flutter, treated or not. Valvulopathy include any cardiac valvular stenosis or insufficiency, mild or severe, with or without cardiac valve replacement. Psychiatric history includes any prior episode important enough to led to medication or hospitalization. Reactive depression was excluded.

All laboratory values were measured using automated and standardized methods that are routinely performed as standard of care. No biological measurements were collected during or after the start of the surgery.

Regarding the imaging variables, indexed cardiac left ventricular mass was manually recorded from the last cardiac echography performed during the year before the kidney transplantation. Transplanted organs were measured during the surgery or assessed during the first imaging modality performed after transplantation by ultrasonography or CT scan.

Donor characteristics
Age
Sex
Size
Weight
Body Mass Index
Hypertension
Diabetes mellitus
Vascular death
Last creatinine
Blood group
Transplant characteristics
Graft rank
Double graft
Number of arteries
Living donation
Donor Specific Antibody
Human Leukocytes Antigen Mismatches
Cold ischemia time
Recipient characteristics
Age
Sex
Size
Weight
Body Mass Index
Chronic Kidney Disease cause
Dialysis and dialysis duration
Dialysis type

© 2026 Debiais-Deschamps C et al. *JAMA Network Open*.

Hypertension
Left Ventricular Hypertrophy
Pregnancy (and number of pregnancies)
Ischemic heart disease
Stroke
Transient ischaemic attack
Peripheral vascular disease
Dyslipidemia
Gout
Diabete mellitus and type
Polycystic disease
Arythmia
Anticoagulation
Valvulopathy or Cardiac valv disorder
Cardiac mechanical valv
Smoking history
Alcohol abuse
Drug abuse
Chronic Obstructive Pulmonary Disease
Asthma
Deep Vein Thrombosis
Pulmonary embolism
Antiphospholipid Antibody Syndrome
Thromboembolic event
Infection
Septicemia
Pyelonephritis
Pneumonia
Tuberculosis
Malaria
Bilharzia
Renal lithiasis
Sickle cell anemia
Peptic Ulcer
Gastritis
Diverticulosis
Pancreatitis
Hepatic Disease
Lupus
Ankylosing spondylitis
Sarcoidosis
Cancer
Hematologic malignancy
Monoclonal gammopathy
Psychiatric history and type
Cognitive disorder

© 2026 Debiais-Deschamps C et al. *JAMA Network Open*.

Epilepsy<br>Thyroid dysfunction<br>Previous organ transplantation (other than kidney)<br>Number of drugs on prescription<br>Serological status against: Cytomegalovirus, Eptein-Barr Virus, Human Immunodeficiency Virus, Hepatitis B Virus, Hepatitis C Virus
Imaging parameters
Length of the kidney<br>Left cardiac ventricular mass
Biological parameters
Glucose<br>Urea<br>Creatinin<br>Albumin<br>Gamma-globulines<br>Protein<br>Ionogramm (Sodium, Potassium, Chloride, Magnesium, Bicarbonate)<br>Troponin<br>Calcium<br>Phosphorus<br>Parathyroid hormone<br>Uric acid<br>Lactate deshydrogenase<br>Iron<br>Ferritin<br>C-Reactive protein<br>HbA1c (glycated haemoglobin)<br>Glutamic-oxaloacetic transaminase<br>Glutamic pyruvic transaminase<br>Alkaline Phosphatase<br>gammaGlutamylTransferase<br>Bilirubin<br>Triglycerids<br>LDL<br>HDL<br>Total Cholesterol<br>Haemoglobin<br>Neutrophils<br>Lymphocytes<br>Activated partial thromboplastin time and Prothrombin Time<br>Vitamin D1-25-OH and Vitamin D-25-OH

© 2026 Debiais-Deschamps C et al. *JAMA Network Open*.

eMethods 6. Management of biological variables for statistical analyses

Variable	Distribution	Proportional hazards assumption	Log-linearity	Transformation	p-value
Glucose	Normal	Yes	Yes	None	<0.001
Urea	Normal	Yes	Yes	None	0.001
Creatinin	Normal	Yes	Yes	None	<0.001
Albumin	Normal	Yes	Yes	None	<0.001
Gamma-globulin	Normal	Yes	Yes	None	0.82
Protein	Normal	Yes	Yes	None	0.15
Na^+	Normal	Yes	Yes	None	0.01
Mg^{2+}	Normal	Yes	Yes	None	0.03
K^+	Normal	Yes	Yes	None	0.12
Cl^-	Normal	Yes	Yes	None	0.05
$HCO3^-$	Normal	Yes	Yes	None	0.26
Troponin	Not normal	Yes	No	Binarized	<0.001
Ca^{2+}	Normal	Yes	Yes	None	0.83
Phosphorus	Normal	Yes	Yes	None	0.02
PTH	Not normal	Yes	Yes	Log transformation	0.06
Uric acid	Normal	Yes	Yes	None	0.03
LDH	Normal	Yes	Yes	None	0.50
Iron	Normal	No	No	Categorized	0.09
Ferritin	Not normal	Yes	No	Log transformation	0.001
C-Reactive Protein	Not normal	No	Yes	Binarized Threshold median	<0.001
HbA1c	Normal	Yes	Yes	None	<0.001
γ-gT	Not normal	Yes	Yes	Log transformation	<0.001
TGO	Normal	Yes	Yes	None	0.006
TGP	Not normal	Yes	Yes	Log transformation	0.13
PAL	Not Normal	Yes	No	Binarized Threshold Q1	0.002
Bilirubin	Normal	Yes	Yes	None	0.35
Triglycerids	Normal	Yes	Yes	None	0.87
LDL	Normal	Yes	Yes	None	0.06
HDL	Normal	Yes	Yes	None	0.76
Total Cholesterol	Normal	Yes	Yes	None	0.56
Haemoglobin	Normal	Yes	Yes	None	0.26
Neutrophils	Normal	Yes	Yes	None	0.11
Lymphocytes	Normal	Yes	Yes	None	0.04
APTT	Normal	Yes	Yes	None	0.31
TP	Normal	Yes	Yes	None	0.004
VitD1-25-OH	Not Normal	Yes	Yes	None	0.60
VitD-25-OH	Normal	Yes	Yes	None	0.01

APTT: Activated Partial Thromboplastin Time; Ca++:Calcium; Cl- :Chloride; CRP: C-reactive Protein; gGT :gammaGlutamylTransferase; HbA1c: Glycated Haemoglobin; HCO3-: Bicarbonate; HDLc: High Density Lipoprotein Cholesterol; K+: Potassium; LDH: Lactate Deshydrogenase; LDLc: Low Density

© 2026 Debiais-Deschamps C et al. *JAMA Network Open*.

Lipoprotein Cholesterol; Mg++: Magnesium; Na+: Sodium; P: Phosphorus; PAL: Alkaline Phosphatase; PTH: Parathyroid Hormone; TGO: Glutamic-oxaloacetic transaminase; TGP: Glutamic pyruvic transaminase; TP: Prothrombin Time

eMethods 7. Penalized regression methods

We performed 2 penalized Cox regressions (LASSO and Elastic Net), allowing for feature selection, to further investigate the agreement between selected variables and variables included in the mBox. 100 repetitions were performed to improve the stability of the selection process. Variables were considered if they were selected more than 60% of the repetitions.

eMethods 8. Construction of the integrative score from the multivariable Cox model

The score was constructed using the linear predictor (sum of beta coefficients) derived from the final Cox model for each patient. mBox predicted probabilities were calculated using the formula $S_0(t)^{exp(X\beta)}$, with $S_0(t)$ being the baseline survival function estimated at the specified time horizon and Xβ being the patient's linear predictor.

$$
\begin{gathered}
\text{Prognostic score}_{\text{mortality}} = \text{Recipient's age} \times \text{a} \\
+ \text{Diabetes history} \binom{0}{1} \times \text{b} \\
+ \text{Valvulopathy} \binom{0}{1} \times \text{c} \\
+ HCVstatus \binom{0}{1} \times \text{d} \\
+ \text{MACCE} \binom{0}{1} \times \text{e} \\
+ \text{Supraventricular cardiac rythm disorder} \binom{0}{1} \times \text{f} \\
+ \text{Psychiatric disorder} \binom{0}{1} \times \text{g} \\
+ \text{Left Ventricular Mass} \times \text{h} \\
+ \text{Kidney transplant size} \binom{0}{1} \times \text{i} \\
+ \text{j1 if dialysis vintage} < \text{3years or j2 if} > \text{3 years} \\
+ \text{Albumin} \times \text{k} \\
+ \text{HbA1c} \times \text{l} \\
+ \text{CRP} > \text{6mg/L} \binom{0}{1} \times \text{m} \\
+ \text{Troponin} > 0.05 \binom{0}{1} \times \text{n} \\
+ \text{Gamma} - \text{GT at 1 Year} \times \text{o}
\end{gathered}
$$

© 2026 Debiais-Deschamps C et al. *JAMA Network Open*.

eMethods 9. Abbreviated mBox models

Model	Number of parameters	Parameters
mBox	14	Age<br>Major cardiovascular event<br>Dialysis duration<br>Valvulopathy<br>Cardiac supraventricular arrythmia<br>Psychiatric history<br>Kidney size<br>Left ventricular mass<br>HCV serology<br>Albumin<br>HbA1c<br>CRP<br>gGT<br>Troponin
Abbreviated model for French validation	10	Age<br>Major cardiovascular event<br>Dialysis duration<br>Valvulopathy<br>Cardiac supraventricular arrythmia<br>HCV serology<br>Albumin<br>HbA1c<br>CRP<br>gGT
Abbreviated model for European validation	7	Age<br>Major cardiovascular event<br>Dialysis duration<br>Albumin<br>HbA1c<br>CRP<br>gGT
Abbreviated model for US validation	11	Age<br>Major cardiovascular event<br>Dialysis duration<br>Valvulopathy<br>Cardiac supraventricular arrythmia<br>Psychiatric history<br>HCV serology<br>Albumin<br>HbA1c<br>CRP<br>gGT
Abbreviated model for GPUH validation (without imaging data)	12	Age<br>Major cardiovascular event<br>Dialysis duration<br>Valvulopathy<br>Cardiac supraventricular arrythmia<br>Psychiatric history<br>HCV serology<br>Albumin<br>Troponin<br>HbA1c<br>CRP<br>gGT
Abbreviated model for UCSF validation	9	Age<br>Major cardiovascular event<br>Dialysis duration<br>Valvulopathy<br>Cardiac supraventricular arrythmia<br>Psychiatric history<br>HCV serology<br>Albumin<br>gGT

© 2026 Debiais-Deschamps C et al. *JAMA Network Open*.

Abbreviated model without cardiac ultrasonography data	13	Age<br>Major cardiovascular event<br>Dialysis duration<br>Valvulopathy<br>Cardiac supraventricular arrythmia<br>Psychiatric history<br>Kidney size<br>HCV serology<br>Albumin<br>HbA1c<br>CRP<br>gGT<br>Troponin
Abbreviated model without imaging data and albumin	11	Age<br>Major cardiovascular event<br>Dialysis duration<br>Valvulopathy<br>Cardiac supraventricular arrythmia<br>Psychiatric history<br>HCV serology<br>HbA1c<br>CRP<br>gGT<br>Troponin
Abbreviated model without biological data	9	Age<br>Major cardiovascular event<br>Dialysis duration<br>Valvulopathy<br>Cardiac supraventricular arrythmia<br>Psychiatric history<br>HCV serology<br>Left ventricular mass<br>Kidney size
Abbreviated model without imaging and biological data	7	Age<br>Major cardiovascular event<br>Dialysis duration<br>Valvulopathy<br>Cardiac supraventricular arrythmia<br>Psychiatric history<br>HCV serology

© 2026 Debiais-Deschamps C et al. *JAMA Network Open*.

eMethods 10. Previously published mortality prediction models

The calculation of the RRS score, the EPTS, the Kasiske score, the KTOP, the iChooseKidney, and the CCI are detailed below.

- RRS score (Baskin-Bey et al.)

RRS =
1.816 if presence of diabetes mellitus
+ 0.448 * age (if no diabetes)
+ 0.213 * age (if presence of diabetes)
+ 0.159 * if dialysis < 1 year + 0.407 * dialysis >1 year
+ 0.303 if angina

- EPTS (Bae et al.)

RawEPTS =
0.047 * max(age-25)
− 0.015 * diabetes * max(age-25)
+ 0.398 * prior solid organ transplant
− 0.237 * diabetes * prior organ transplant
+ 0.315 * log(years on Dialysis + 1) − 0.099 * diabetes * log(years on dialysis + 1)
+ 0.130 * (years on dialysis = 0) − 0.348 * diabetes * (years on dialysis = 0) + 1.262 * diabetes

- Model by Kasiske et al.

Kasiske score =
0.0088 * (donor age-38) + 0.0003 * (Donor age-38)^2
+ dialysis * (-0.2883 if preemptive transplantation, -0.0797 if dialysis <1 year, 0.0224 if dialysis >3 years and <5 years and 0.1565 if dialysis >5 years; 0.3815 if dialysis <9 years and retransplantation, 0.3392 if dialysis between 8 and 14 years and retransplantation and 0.2631 if dialysis >14 years and retransplantation)
+ 0.0089 * (recipient age-50)
+ 0.0006 * (recipient age-50)^2
-0.0001*(recipient age-50) * (Donor Age-38)
-0.2097(or according to initial nephropathy: 0 if diabetes, -0.1740 if hypertension, -0.2555 if glomerulonephritis, -0.5202 if cystic disease)
+ 0.3978 * HCV
+ 0.2009 * Donor hypertension
-0.1623 if trauma as cause of donor death
+ 0.1466 if 1 to 3 HLA mismatches
+ 0.2376 if 4 to 6 HLA mismatches

- KTOP (Miller et al.)

KTOP =
log(1.08) * recipient age
+ log(1.01) * donor age
+ log(10.21) * 0.97^(recipient age) if diabetes
+ log(0.64) if PKRAD (change for 1.28 if male)
+ (transplantation rank -1) * log(2.81) * 0.9^(recipient age)
+ log(1.06) * waiting time

- iChooseKidney (Patzer et al.)

iChooseKidney =
-5.4292
+ (-0.0475 if female recipient)
+ 0.0382 * recipient age
+ 0.3369 * cardiovascular disease
- 0.2000 * hypertension
+ 0.4013 * diabetes
+ 0.2102 if albumin <3.5 g/dL
+ (0.1360 if dialysis duration between 6-12 months or 0.4906 if dialysis duration > 12 months)

- CCI (Charlson et al.)

CCI = 0.983^(exp(comorbidity))

Where comorbidity is the sum of weighted index of conditions:

**Assigned weights for diseases**	**Conditions**
1	Myocardial infarct<br>Congestive heart failure<br>Peripheral vascular disease<br>Cerebrovascular disease<br>Dementia<br>Chronic pulmonary disease<br>Ulcer disease<br>Mild liver disease<br>Diabetes
2	Hemiplegia<br>Moderate or severe renal disease<br>Diabetes with end organ damage<br>Any tumor<br>Leukemia<br>Lymphoma
3	Moderate or severe liver disease
6	Metastatic solid tumor<br>AIDS

In our cohort, no patient had metastatic solid tumor, moderate or severe liver disease or hemiplegia. All had at least terminal renal disease.

© 2026 Debiais-Deschamps C et al. *JAMA Network Open*.

eMethods 11. Machine Learning models

We investigated Machine Learning (ML) survival models, i.e., ML models that can be applied to time-to-event data, using the following approaches: Random Survival Forest (RSF), Random Survival Forest with Extremely Randomized Trees (RSF-ERT), Random Survival Forest with Maximally Selected Rank Statistics (RSF-MaxStat), Gradient Boosting with Cox model base learner (GBM-Cox), Gradient Boosting with decision tree base learner (GBM-DecisionTree), and Extreme Gradient Boosting (XGBoost). For models that rely on the Cox partial likelihood as their loss function (GBM-Cox, GBM-DecisionTree, and XGBoost), the same functional forms of variables as in the mBox model were used.

The derivation cohort was split into a 70/30% training/test set, with stratification to maintain the same distribution of event times and outcomes in each subset. For each model, hyperparameters were optimized using 5-fold cross-validation with a grid search strategy on the training set. The optimal combination of hyperparameters was selected by maximizing the C-statistic across the 5 folds. The hyperparameters tested for each model are reported in the table below.

**Machine Learning model**	**Hyperparameters**
Random Survival Forest	mtry, nsplit, nodesize, ntree, splitrule
Random Survival Forest with Extremely Randomized Trees	mtry, nodesize, ntree, splitrule
Random Survival Forest with Maximally Selected Rank Statistics	mtry, nodesize, ntree, alpha
Gradient Boosting with Cox model base learner	mstop, $\boldsymbol{\nu}$ parameter
Gradient Boosting with decision tree base learner	n.trees, shrinkage, interaction.depth, n.minobsinnode, bag.fraction
Extreme Gradient Boosting (XGBoost)	nrounds, eta, max_depth, min_child_weight, subsample, colsample_bytree, $\boldsymbol{\alpha}$, $\boldsymbol{\lambda}$, $\boldsymbol{\gamma}$ parameters

Model performance was reported on the held-out test set, with 95% confidence intervals derived using the bias-corrected and accelerated (BCa) bootstrap method. To identify the main determinants of recipients' death, we evaluated variable importance for all models except RSF-ERT.

© 2026 Debiais-Deschamps C et al. *JAMA Network Open*.

TABLES

eTable 1. Baseline characteristics of the derivation cohort before and after missing data imputation

	Before imputation		After imputation	
	n		n	
**Donor characteristics**				
Age, mean (SD), years	1566	54.24 (17.00)	1566	54.24 (17.00)
Gender male, No. (%)	1566	816 (52.11)	1566	816 (52.11)
Body Mass Index, mean (SD), kg/m2	1566	25.55 (4.99)	1566	25.55 (4.99)
Donor height, mean (SD), cm	1566	168.96 (10.91)	1566	168.96 (10.91)
Donor weight, mean (SD), kg	1566	73.25 (16.30)	1566	73.25 (16.30)
Hypertension, No. (%)	1566	466 (29.76)	1566	466 (29.76)
Diabete mellitus, No. (%)	1564	120 (7.67)	1566	120 (7.66)
Last creatinine, mean (SD), µmol/L	1566	88.61 (51.12)	1566	88.61 (51.12)
**Transplant characteristics**				
Graft rank, No. (%)	1566		1566	
1		1272 (81.2)		1272 (81.23)
2		226 (14.4)		226 (14.43)
3		60 (3.83)		60 (3.83)
4		6 (0.38)		6 (0.38)
5		2 (0.13)		2 (0.13)
Double graft, No. (%)	1532	146 (9.53)	1566	147 (9.39)
More than one artery, No. (%)	1514	363 (23.97)	1566	376 (24.01)
Living Donation, No. (%)	1566	346 (22.09)	1566	346 (22.09)
DSA, No. (%)	1566	337 (21.52)	1566	337 (21.52)
HLA A/B/DR mismatch, mean (SD), number	1565	3.62 (1.40)	1566	3.63 (1.40)
Cold ischemia time, median (IQR), min	1560	1080 (673 -1480)	1566	1080 (675 - 1474)
**Recipient characteristics**				
Age, mean (SD), years	1566	50.05 (14.31)	1566	50.05 (14.31)
Gender male, No. (%)	1566	942 (60.15)	1566	942 (60.15)
Body Mass Index	1560	23.82 (4.44)	1566	23.82 (4.43)
ESRD causes	1566		1566	
Glomerulonephritis, No. (%)		440 (28.10)		440 (28.10)
PKD, No. (%)		151 (9.64)		151 (9.64)
Diabetes, No. (%)		133 (8.49)		133 (8.49)
Hypertension, No. (%)		69 (4.41)		69 (4.41)
Other, No. (%)		773 (49.36)		773 (49.36)
Dialysis, No. (%)	1537	1245 (81.00)	1566	1274 (81.35)
Time since onset of dialysis, median (IQR), years	1534	3.19 (0.87-6.37)	1566	3.32 (0.90-6.46)
**Imaging**				
Cardiac Echography				
Left Ventricular Mass, mean (SD)	976	128.39 (36.20)	1566	129.04 (29.21)

© 2026 Debiais-Deschamps C et al. *JAMA Network Open*.

Transplant Echography				
Kidney Size, mean (SD), cm	1382	11.03 (0.94)	1566	11.03 (0.89)
**Comorbidities**				
Hypertension, No. (%)	1536	1158 (75.39)	1566	1187 (75.80)
Left ventricular hypertrophy, No. (%)	1366	249 (18.23)	1566	250 (15.96)
Ischemic heart disease, No. (%)	1534	193 (12.58)	1566	193 (12.32)
Stroke, No. (%)	1566	54 (3.45)	1566	54 (3.45)
Peripheral vascular disease, No. (%)	1535	61 (3.97)	1566	61 (3.90)
MACCE, No. (%)	1535	253 (16.48)	1566	253 (16.16)
Dyslipidemia, No. (%)	1533	589 (38.42)	1566	599 (38.25)
Gout, No. (%)	1366	78 (5.71)	1566	78 (4.98)
Diabete mellitus, No. (%)	1536	215 (14.00)	1566	218 (13.92)
Atrial arythmia, No. (%)	1536	76 (4.95)	1566	76 (4.85)
Cardiac Valv Disorder, No. (%)	1366	61 (4.47)	1566	61 (3.90)
Mechanical valv, No. (%)	1366	15 (1.10)	1566	15 (0.96)
Smoking, No. (%)	1512	482 (31.88)	1566	485 (30.97)
COPD, No. (%)	1535	32 (2.08)	1566	32 (2.04)
Asthma, No. (%)	1536	57 (3.71)	1566	57 (3.64)
Alcohol abuse, No.(%)	1533	86 (5.61)	1566	86 (5.49)
Drug Abuse, No. (%)	1367	6 (0.44)	1566	6 (0.38)
Deep Vein Thrombosis, No. (%)	1536	94 (6.12)	1566	94 (6.00)
Pulmonary embolism, No. (%)	1536	34 (2.21)	1566	34 (2.17)
APLS, No. (%)	1535	19 (1.24)	1566	19 (1.21)
Thromboembolic event, No. (%)	1430	106 (6.90)	1566	106 (6.77)
Infection, No. (%)	1335	470 (35.21)	1566	501 (31.99)
Septicemia, No. (%)	1366	67 (4.90)	1566	67 (4.28)
Pyelonephritis, No. (%)	1535	69 (4.50)	1566	69 (4.41)
Pneumonia, No. (%)	1366	62 (4.54)	1566	62 (3.86)
Tuberculosis, No. (%)	1534	74 (4.82)	1566	74 (4.73)
Sarcoidosis, No. (%)	1367	5 (0.51)	1566	7 (0.45)
Malaria, No. (%)	1366	33 (2.42)	1566	33 (2.11)
Bilharzia, No. (%)	1366	16 (1.17)	1566	16 (1.02)
Renal Colic, No. (%)	1366	33 (2.42)	1566	33 (2.11)
Sickle cell anemia, No. (%)	1365	22 (1.61)	1566	22 (1.40)
Peptic Ulcer, No. (%)	1366	97 (7.10)	1566	97 (6.19)
Gastritis, No. (%)	1366	69 (5.05)	1566	69 (4.41)
Diverticulosis, No. (%)	1366	52 (3.81)	1566	52 (3.32)
Pancreatitis, No. (%)	1366	24 (1.76)	1566	24 (1.53)
Hepatic Disease, No. (%)	1366	35 (2.56)	1566	35 (2.23)
Lupus, No. (%)	1366	34 (2.49)	1566	34 (2.17)
PKD, No. (%)	1566	151 (9.64)	1566	151 (9.64)
Glomerulonephritis, No. (%)	1566	421 (28.10)	1566	421 (28.10)
CIN, No. (%)	1566	237 (15.13)	1566	237 (15.13)
Vascular nephropathy, No. (%)	1566	88 (5.62)	1566	88 (5.62)
Cancer, No. (%)	1536	120 (7.81)	1566	133 (8.49)
Hematologic malignancy, No. (%)	1536	14 (0.91)	1566	14 (0.89)

© 2026 Debiais-Deschamps C et al. *JAMA Network Open*.

Monoclonal gammopathy, No. (%)	1535	27 (1.76)	1566	27 (1.72)
Psychiatric history, No. (%)	1535	89 (5.80)	1566	89 (5.68)
Thyroid dysfunction, No. (%)	1322	44 (3 .22)	1566	44 (2.81)
Organ Graft (other than kidney), No. (%)	1566	24 (1.53)	1566	24 (1.53)
Number of drugs, mean (SD)	1351	7.39 (3.05)	1566	7.37 (2.85)
Anticoagulation, No. (%)	1532	78 (5.09)	1566	78 (4.98)
Pregnancy, No. (%)	1536	385 (25.07)	1566	401 (25.61)
CMV, No. (%)	1557	1,132 (72.70)	1566	1,141 (72.86)
EBV, No. (%)	1557	1,479 (94.99)	1566	1,488 (95.02)
VIH, No. (%)	1553	29 (1.89)	1566	29 (1.85)
HBV, No. (%)	1454	273 (18.78)	1566	274 (17.50)
HCV, No. (%)	1548	96 (6.20)	1566	96 (6.13)

Cancer includes solid cancer even cutaneous ones, lymphoma and leukemia. DSA: Donor Specific Antibodies; ESRD: End Stage Renal Disease; MACCE: Major Cardio-Vascular Event, combined criteria including coronaropathy, myocardial infarction and stroke. COPD: Chronic Obstructive Pulmonary Disease; APLS: Antiphospholipid Antibody Syndrome; PKD: Polycystic Kidney Disease; CIN: Chronic Interstitial Nephropathy; HBV: Hepatitis B Virus (HBV infection past or present, excluding vaccination only); HCV: Hepatitis C Virus

© 2026 Debiais-Deschamps C et al. *JAMA Network Open*.

eTable 2. Baseline characteristics of the French validation cohort

	SLS-Bichat (N=827)	Toulouse (N=331)	Tours (N=495)	Overall (N=1653)
Recipient Age				
Mean (SD)	50.2 (13.4)	54.6 (15.4)	56.4 (14.7)	52.9 (14.5)
Recipient Sex				
Male	526 (63.6%)	206 (62.2%)	332 (67.1%)	1064 (64.4%)
Female	301 (36.4%)	125 (37.8%)	163 (32.9%)	589 (35.6%)
Recipient BMI				
Mean (SD)	24.0 (4.53)	25.3 (4.69)	26.2 (5.54)	25.0 (4.97)
Missing	4 (0.5%)	1 (0.3%)	8 (1.6%)	13 (0.8%)
Initial Nephropathy				
GN	193 (23.3%)	91 (27.5%)	115 (23.2%)	399 (24.1%)
Diabetes	106 (12.8%)	43 (13.0%)	62 (12.5%)	211 (12.8%)
Other	94 (11.4%)	85 (25.7%)	76 (15.4%)	255 (15.4%)
Polycystic	93 (11.2%)	38 (11.5%)	69 (13.9%)	200 (12.1%)
TIN	72 (8.7%)	30 (9.1%)	11 (2.2%)	113 (6.8%)
Vascular	106 (12.8%)	22 (6.6%)	30 (6.1%)	158 (9.6%)
Unknown	163 (19.7%)	22 (6.6%)	132 (26.7%)	317 (19.2%)
DVA				
Presence	138 (16.7%)	99 (29.9%)	53 (10.7%)	290 (17.5%)
Absence	689 (83.3%)	232 (70.1%)	442 (89.3%)	1363 (82.5%)
Transplantation rank				
1	718 (86.8%)	280 (84.6%)	411 (83.0%)	1409 (85.2%)
2	99 (12.0%)	38 (11.5%)	70 (14.1%)	207 (12.5%)
3	9 (1.1%)	13 (3.9%)	12 (2.4%)	34 (2.1%)
4	1 (0.1%)	0 (0%)	2 (0.4%)	3 (0.2%)
Donor Age				
Mean (SD)	51.0 (15.7)	56.8 (15.8)	57.4 (17.5)	54.1 (16.6)
Donor BMI				
Mean (SD)	25.4 (4.53)	25.8 (5.21)	26.3 (5.29)	25.7 (4.92)
Missing	2 (0.2%)	0 (0%)	0 (0%)	2 (0.1%)

© 2026 Debiais-Deschamps C et al. *JAMA Network Open*.

	SLS-Bichat (N=827)	Toulouse (N=331)	Tours (N=495)	Overall (N=1653)
Diabetes				
Presence	179 (21.6%)	70 (21.1%)	126 (25.5%)	375 (22.7%)
Absence	644 (77.9%)	261 (78.9%)	369 (74.5%)	1274 (77.1%)
Missing	4 (0.5%)	0 (0%)	0 (0%)	4 (0.2%)
MACCE				
Presence	184 (22.2%)	87 (26.3%)	116 (23.4%)	387 (23.4%)
Absence	638 (77.1%)	244 (73.7%)	375 (75.8%)	1257 (76.0%)
Missing	5 (0.6%)	0 (0%)	4 (0.8%)	9 (0.5%)
HCV serology				
Positive	48 (5.8%)	6 (1.8%)	13 (2.6%)	67 (4.1%)
Negative	778 (94.1%)	318 (96.1%)	482 (97.4%)	1578 (95.5%)
Missing	1 (0.1%)	7 (2.1%)	0 (0%)	8 (0.5%)
Cardiac Valv disorder				
Presence	76 (9.2%)	8 (2.4%)	96 (19.4%)	180 (10.9%)
Absence	751 (90.8%)	323 (97.6%)	393 (79.4%)	1467 (88.7%)
Missing	0 (0%)	0 (0%)	6 (1.2%)	6 (0.4%)
Cardiac rythm disorder				
Presence	69 (8.3%)	31 (9.4%)	58 (11.7%)	158 (9.6%)
Absence	758 (91.7%)	300 (90.6%)	433 (87.5%)	1491 (90.2%)
Missing	0 (0%)	0 (0%)	4 (0.8%)	4 (0.2%)
Psychiatric history				
Presence	14 (1.7%)	0 (0%)	29 (5.9%)	43 (2.6%)
Absence	812 (98.2%)	0 (0%)	466 (94.1%)	1278 (77.3%)
Missing	1 (0.1%)	331 (100%)	0 (0%)	332 (20.1%)
HbA1C (%)				
Mean (SD)	5.72 (1.11)	5.70 (1.13)	5.70 (0.926)	5.71 (1.05)
Missing	95 (11.5%)	152 (45.9%)	19 (3.8%)	266 (16.1%)
gGT (UI/L)				
Median [Min, Max]	22.0 [5.00, 510]	22.0 [4.00, 189]	25.0 [5.00, 1860]	23.0 [4.00, 1860]
Missing	17 (2.1%)	46 (13.9%)	2 (0.4%)	65 (3.9%)

© 2026 Debiais-Deschamps C et al. *JAMA Network Open*.

	SLS-Bichat (N=827)	Toulouse (N=331)	Tours (N=495)	Overall (N=1653)
Troponin (ng/mL)				
Median [Min, Max]	0.0224 [0.0100, 0.704]	NA	NA	0.0224 [0.0100, 0.704]
Missing	66 (8.0%)	331 (100%)	495 (100%)	892 (54.0%)
CRP (mg/L)				
Median [Min, Max]	2.00 [1.90, 97.0]	2.70 [0.0600, 209]	3.10 [0.300, 115]	2.90 [0.0600, 209]
Missing	23 (2.8%)	72 (21.8%)	3 (0.6%)	98 (5.9%)
Albumin (g/L)				
Mean (SD)	42.3 (4.59)	39.4 (5.77)	42.0 (4.31)	41.8 (4.78)
Missing	71 (8.6%)	119 (36.0%)	4 (0.8%)	194 (11.7%)
Vital Status				
Dead	209 (25.3%)	27 (8.2%)	91 (18.4%)	327 (19.8%)
Alive	618 (74.7%)	304 (91.8%)	404 (81.6%)	1326 (80.2%)
Survival time (years)				
Median [Min, Max]	8.50 [0.00274, 13.9]	1.70 [0.00100, 5.37]	5.83 [0.0219, 8.21]	6.28 [0.00100, 13.9]

© 2026 Debiais-Deschamps C et al. *JAMA Network Open*.

eTable 3. Baseline characteristics of the European validation cohort

	**Leiden (N=1328)**	**Liege (N=609)**	**Leuven (N=1890)**	**Overall (N=3827)**
Recipient Age				
Mean (SD)	54.2 (13.7)	52.5 (13.2)	54.6 (12.8)	54.1 (13.2)
Recipient Sex				
Male	836 (63.0%)	381 (62.6%)	1198 (63.4%)	2415 (63.1%)
Female	492 (37.0%)	228 (37.4%)	692 (36.6%)	1412 (36.9%)
Recipient BMI				
Mean (SD)	25.6 (4.14)	26.4 (5.11)	25.6 (4.52)	25.7 (4.46)
Missing	84 (6.3%)	226 (37.1%)	0 (0%)	310 (8.1%)
Initial Nephropathy				
GN	467 (35.2%)	0 (0%)	608 (32.2%)	1075 (28.1%)
Diabetes	220 (16.6%)	0 (0%)	198 (10.5%)	418 (10.9%)
Other	344 (25.9%)	0 (0%)	238 (12.6%)	582 (15.2%)
Polycystic	185 (13.9%)	0 (0%)	330 (17.5%)	515 (13.5%)
TIN	43 (3.2%)	0 (0%)	193 (10.2%)	236 (6.2%)
Vascular	39 (2.9%)	0 (0%)	109 (5.8%)	148 (3.9%)
Unknown	30 (2.3%)	0 (0%)	214 (11.3%)	244 (6.4%)
Missing	0 (0%)	609 (100%)	0 (0%)	609 (15.9%)
DVA				
Presence	742 (55.9%)	42 (6.9%)	147 (7.8%)	931 (24.3%)
Absence	586 (44.1%)	567 (93.1%)	1743 (92.2%)	2896 (75.7%)
Transplantation rank				
1	1161 (87.4%)	535 (87.8%)	1606 (85.0%)	3302 (86.3%)
2	154 (11.6%)	67 (11.0%)	232 (12.3%)	453 (11.8%)
3	12 (0.9%)	7 (1.1%)	42 (2.2%)	61 (1.6%)
4	1 (0.1%)	0 (0%)	9 (0.5%)	10 (0.3%)
5	0 (0%)	0 (0%)	1 (0.1%)	1 (0.0%)
Donor Age				
Mean (SD)	54.0 (12.8)	44.3 (14.2)	49.1 (14.3)	50.1 (14.2)
Missing	7 (0.5%)	0 (0%)	0 (0%)	7 (0.2%)
Donor BMI				

© 2026 Debiais-Deschamps C et al. *JAMA Network Open*.

	Leiden (N=1328)	Liege (N=609)	Leuven (N=1890)	Overall (N=3827)
Mean (SD)	25.7 (3.82)	25.2 (4.63)	25.2 (4.00)	25.4 (4.05)
Missing	17 (1.3%)	1 (0.2%)	51 (2.7%)	69 (1.8%)
Diabetes				
Presence	298 (22.4%)	143 (23.5%)	344 (18.2%)	785 (20.5%)
Absence	894 (67.3%)	465 (76.4%)	1546 (81.8%)	2905 (75.9%)
Missing	136 (10.2%)	1 (0.2%)	0 (0%)	137 (3.6%)
MACCE				
Presence	400 (30.1%)	103 (16.9%)	578 (30.6%)	1081 (28.2%)
Absence	783 (59.0%)	506 (83.1%)	1312 (69.4%)	2601 (68.0%)
Missing	145 (10.9%)	0 (0%)	0 (0%)	145 (3.8%)
HCV serology				
Positive	11 (0.8%)	14 (2.3%)	0 (0%)	25 (0.7%)
Negative	1047 (78.8%)	1 (0.2%)	1890 (100%)	2938 (76.8%)
Missing	270 (20.3%)	594 (97.5%)	0 (0%)	864 (22.6%)
Cardiac Valv disorder				
Presence	0 (0%)	8 (1.3%)	12 (0.6%)	20 (0.5%)
Absence	0 (0%)	601 (98.7%)	1878 (99.4%)	2479 (64.8%)
Missing	1328 (100%)	0 (0%)	0 (0%)	1328 (34.7%)
Cardiac rythm disorder				
Presence	0 (0%)	0 (0%)	138 (7.3%)	138 (3.6%)
Absence	0 (0%)	0 (0%)	1752 (92.7%)	1752 (45.8%)
Missing	1328 (100%)	609 (100%)	0 (0%)	1937 (50.6%)
Psychiatric history				
Presence	0 (0%)	0 (0%)	332 (17.6%)	332 (8.7%)
Absence	0 (0%)	0 (0%)	1558 (82.4%)	1558 (40.7%)
Missing	1328 (100%)	609 (100%)	0 (0%)	1937 (50.6%)
HbA1C (%)				
Mean (SD)	5.65 (1.05)	5.45 (0.974)	5.50 (0.785)	5.54 (0.919)
Missing	15 (1.1%)	71 (11.7%)	98 (5.2%)	184 (4.8%)
gGT (UI/L)				
Median [Min, Max]	22.0 [4.00, 608]	21.0 [5.00, 619]	22.0 [5.00, 570]	22.0 [4.00, 619]
Missing	13 (1.0%)	88 (14.5%)	77 (4.1%)	178 (4.7%)

© 2026 Debiais-Deschamps C et al. *JAMA Network Open*.

	Leiden (N=1328)	Liege (N=609)	Leuven (N=1890)	Overall (N=3827)
Troponin (ng/mL)				
Median [Min, Max]	NA	NA	0.0300 [0.00400, 3.83]	0.0300 [0.00400, 3.83]
Missing	1328 (100%)	609 (100%)	199 (10.5%)	2136 (55.8%)
CRP (mg/L)				
Median [Min, Max]	3.00 [0.300, 299]	3.00 [0.100, 386]	3.00 [0.100, 84.3]	3.00 [0.100, 386]
Missing	894 (67.3%)	34 (5.6%)	104 (5.5%)	1032 (27.0%)
Albumin (g/L)				
Mean (SD)	38.6 (5.15)	41.9 (6.10)	43.5 (4.63)	41.4 (5.57)
Missing	8 (0.6%)	74 (12.2%)	326 (17.2%)	408 (10.7%)
Vital Status				
Dead	244 (18.4%)	201 (33.0%)	593 (31.4%)	1038 (27.1%)
Alive	1084 (81.6%)	408 (67.0%)	1297 (68.6%)	2789 (72.9%)
Survival time (years)				
Median [Min, Max]	5.00 [0.0833, 11.0]	8.44 [0.00548, 15.9]	6.41 [0.00100, 18.2]	6.00 [0.00100, 18.2]

© 2026 Debiais-Deschamps C et al. *JAMA Network Open*.

eTable 4. Baseline characteristics of the US validation cohort

	**Mayo (N=500)**	**Philadelphia (N=383)**	**Overall (N=883)**
Recipient Age			
Mean (SD)	54.5 (14.2)	50.7 (13.6)	52.9 (14.0)
Recipient Sex			
Male	282 (56.4%)	205 (53.5%)	487 (55.2%)
Female	218 (43.6%)	178 (46.5%)	396 (44.8%)
Recipient BMI			
Mean (SD)	28.3 (5.66)	27.4 (5.86)	27.9 (5.76)
Initial Nephropathy			
GN	87 (17.4%)	109 (28.5%)	196 (22.2%)
Diabetes	155 (31.0%)	67 (17.5%)	222 (25.1%)
Other	97 (19.4%)	43 (11.2%)	140 (15.9%)
Polycystic	39 (7.8%)	42 (11.0%)	81 (9.2%)
TIN	12 (2.4%)	6 (1.6%)	18 (2.0%)
Vascular	55 (11.0%)	81 (21.1%)	136 (15.4%)
Unknown	55 (11.0%)	33 (8.6%)	88 (10.0%)
Missing	0 (0%)	2 (0.5%)	2 (0.2%)
DVA			
Presence	97 (19.4%)	111 (29.0%)	208 (23.6%)
Absence	403 (80.6%)	271 (70.8%)	674 (76.3%)
Missing	0 (0%)	1 (0.3%)	1 (0.1%)
Transplantation rank			
1	442 (88.4%)	325 (84.9%)	767 (86.9%)
2	51 (10.2%)	48 (12.5%)	99 (11.2%)
3	6 (1.2%)	8 (2.1%)	14 (1.6%)
4	1 (0.2%)	2 (0.5%)	3 (0.3%)
Donor Age			
Mean (SD)	40.5 (15.7)	37.0 (15.3)	39.0 (15.6)
Missing	0 (0%)	1 (0.3%)	1 (0.1%)
Donor BMI			
Mean (SD)	29.7 (8.46)	26.7 (6.33)	28.2 (7.64)

© 2026 Debiais-Deschamps C et al. *JAMA Network Open*.

	Mayo (N=500)	Philadelphia (N=383)	Overall (N=883)
Missing	102 (20.4%)	0 (0%)	102 (11.6%)
Diabetes			
Presence	195 (39.0%)	109 (28.5%)	304 (34.4%)
Absence	305 (61.0%)	274 (71.5%)	579 (65.6%)
MACCE			
Presence	105 (21.0%)	80 (20.9%)	185 (21.0%)
Absence	395 (79.0%)	303 (79.1%)	698 (79.0%)
HCV serology			
Positive	29 (5.8%)	31 (8.1%)	60 (6.8%)
Negative	471 (94.2%)	349 (91.1%)	820 (92.9%)
Missing	0 (0%)	3 (0.8%)	3 (0.3%)
Cardiac Valv disorder			
Presence	39 (7.8%)	43 (11.2%)	82 (9.3%)
Absence	461 (92.2%)	340 (88.8%)	801 (90.7%)
Cardiac rythm disorder			
Presence	48 (9.6%)	28 (7.3%)	76 (8.6%)
Absence	452 (90.4%)	355 (92.7%)	807 (91.4%)
Psychiatric history			
Presence	50 (10.0%)	70 (18.3%)	120 (13.6%)
Absence	450 (90.0%)	312 (81.5%)	762 (86.3%)
Missing	0 (0%)	1 (0.3%)	1 (0.1%)
HbA1C (%)			
Mean (SD)	6.06 (1.44)	5.50 (1.31)	5.80 (1.41)
Missing	267 (53.4%)	189 (49.3%)	456 (51.6%)
Albumin (g/L)			
Mean (SD)	43.0 (4.25)	40.2 (7.32)	42.3 (5.30)
Missing	1 (0.2%)	223 (58.2%)	224 (25.4%)
Vital Status			
Dead	78 (15.6%)	45 (11.7%)	123 (13.9%)
Alive	422 (84.4%)	338 (88.3%)	760 (86.1%)

© 2026 Debiais-Deschamps C et al. *JAMA Network Open*.

	Mayo (N=500)	Philadelphia (N=383)	Overall (N=883)
Survival time (years)			
Median [Min, Max]	6.01 [0.0767, 7.18]	6.79 [0.00548, 8.48]	6.40 [0.00548, 8.48]

© 2026 Debiais-Deschamps C et al. *JAMA Network Open*.

eTable 5. Baseline characteristics of the clinical data warehouses validation cohorts

	n	GPUH cohort (n=2,560)	n	UCSF cohort (n=2,028)
**Recipient demographics**				
Age (years), mean (SD)	2,560	53.06 (14.75)	2,028	52.68 (13.87)
Gender male, No. (%)	2,560	1,610 (62.89)	2,028	1,190 (58,68)
Dialysis, No. (%)	2,560	1,210 (47.27)	2,028	1,384 (68,24)
**Recipient history**				
MACE PAD, No. (%)	2,560	486 (18.98)	2,028	327 (16.12)
Atrial arythmia, No. (%)	2,560	97 (3.79)	2,028	101 (4.98)
Cardiac Valv disorder, No. (%)	2,560	67 (2.62)	2,028	66 (3.25)
Psychiatric history, No. (%)	2,560	97 (3.79)	2,028	40 (1.97)
HCV, No. (%)	2,560	69 (2.70)	2,028	33 (1.63)
Albumin (g/dL), mean (SD)	-	-	2,028	36.27 (4.38)
HbA1c (%), mean (SD)	2,560	5.66 (0.94)	2,028	5.83 (0.79)
Troponin positivity, No. (%)	2,560	890 (34.77)	-	-
CRP positivity, No. (%)	2,560	1,355 (52.93)	2,028	1,279 (63.07)
gGT, median (IQR)	2,560	25 (16-39)	2,028	24 (16-37)

© 2026 Debiais-Deschamps C et al. *JAMA Network Open*.

eTable 6. Cox univariable analyses

	Variable		Number of patients	Number of events	HR	95% CI	p-value
**Baseline recipient characteristics**	Age (per 1-yr increment)		1566	414	1.08	(1.07 to 1.09)	<0.001
	Gender	Female	624	156	1	-	
		Male	942	258	1.12	(0.92 to 1.36)	0.28
	Body Mass Index		1566	414	1.06	(1.03 to 1.08)	<0.001
	Dialysis	No	292	50	1	-	
		Yes, <3 years	450	123	1.71	(1.23 to 2.37)	0.001
		Yes, >3 years	824	241	1.83	(1.35 to 2.49)	<0.001
	Initial nephropathy	Glomerular	421	85	1	-	
		Diabetes	133	72	3.38	(2.47 to 4.63)	<0.001
		NIC	237	49	1.03	(0.72 to 1.46)	0.88
		Vascular	88	37	2.53	(1.72 to 3.72)	<0.001
		Cystic disease	151	34	1.13	(0.76 to 1.69)	0.54
		Toxic	18	9	3.33	(1.58 to 6.62)	<0.001
		Unknown	334	85	1.28	(0.95 to 1.73)	0.11
		Other	184	43	1.17	(0.81 to 1.69)	0.40
	CMV status	Negative	425	87	1	-	0.003
		Positive	1141	327	1.44	(1.35 to 1.82)	
	EBV status	Negative	78	23	1	-	0.47
		Positive	1488	391	0.86	(0.56 to 1.31)	
	HIV status	Negative	1129	408	1	-	0.55
		Positive	23	6	0.78	(0.35 to 1.75)	
	HBV status	Negative	1292	334	1	-	0.28
		Positive	274	80	1.14	(0.90 to 1.46)	
	VHC status	Negative	1470	375	1	-	
		Positive	96	39	1.74	(1.25 to 2.41)	0.001
**Baseline donor characteristics**	Age (per 1-yr increment)		1566	414	1.04	(1.04 to 1.05)	<0.001
	Gender	Female	750	219	1	-	
		Male	816	195	0.78	(0.64 to 0.95)	0.01
	Donor weight (per kg increment)		1566	414	1.002	1.00 to 1.01)	0.58
	Donor height, (per cm increment)		1566	414	0.99	(0.98 to 0.99)	<0.001
	Body Mass Index		1566	414	1.03	(1.01 to 1.05)	<0.001
	Last creatinin		1566	414	0.998	(1.00 to 1.00)	0.131
	Living donor	No	1220	376	1	-	
		Yes	346	38	0.32	(0.23 to 0.45)	<0.001

© 2026 Debiais-Deschamps C et al. *JAMA Network Open*.

	CV death	No	502	112	1	-	
		Yes	718	264	1.83	(1.47 to 2.28)	<0.001
	Hypertension	No	1100	237	1	-	
		Yes	466	177	1.995	(1.64 to 2.42)	<0.001
	Diabetes	No	1446	367	1	-	
		Yes	120	47	1.70	(1.26 to 2.31)	<0.001
**Baseline transplant characteristics**	Graft rank	1	1272	335	1	-	
		>1	294	79	1.01	(0.79 to 1.29)	0.94
	Double graft	No	1419	333	1	-	
		Yes	147	81	2.94	(2.31 to 3.75)	<0.001
	Number of arteries	1	1190	304	1	-	
		>1	376	110	1.21	(0.97 to 1.50)	0.09
	Cold ischemia time	≤720 min	429	59	1	-	
		>720 min	1137	355	2.48	(1.89 to 3.27)	<0.001
**Immunological parameters**	No. of HLA A/B/DR mismatches		1566	414	1.17	(1.09 to 1.26)	<0.001
	Anti-HLA DSA on day 0	No	1229	315	1	-	
		Yes	337	99	1.19	(0.95 to 1.49)	0.14
**Comorbidities**	Hypertension	No	382	78	1	-	
		Yes	1184	336	1.45	(1.13 to 1.85)	0.003
	Diabetes	No	1348	298	1	-	
		Yes	218	116	3.03	(2.44 to 3.75)	<0.001
	Coronaropathy	No	1373	311	1	-	
		Yes	193	103	2.93	(2.35 to 3.67)	<0.001
	Stroke	No	1512	386	1	-	
		Yes	54	28	2.51	(1.71 to 3.69)	<0.001
	PAD	No	1505	374	1	-	
		Yes	61	40	3.66	(2.64 to 5.07)	<0.001
	MACCE	No	1315	281	1	-	<0.001
		Yes	251	133	3.12	(2.54 to 3.84)	
	Dyslipidemia	No	967	185	1	-	
		Yes	599	229	2.28	(1.87 to 2.76)	<0.001
	Gout	No	1488	391	1	-	0.32
		Yes	78	23	1.24	(0.81 to 1.88)	
	Smoking	No	1081	248	1	-	
		Yes	485	166	1.60	(1.31 to 1.95)	<0.001
	COPD	No	1534	395	1	-	
		Yes	32	19	3.19	(2.01 to 5.06)	<0.001
	Asthma	No	1509	409	1	-	
		Yes	57	5	0.29	(0.12 to 0.70)	0.006
	Deep Vein Thrombosis	No	1472	383	1	-	
		Yes	94	31	1.39	(0.96 to 2.00)	0.078
	Pulmonary Embolism	No	1532	400	1	-	
		Yes	34	14	1.80	(1.06 to 3.07)	0.03

© 2026 Debiais-Deschamps C et al. *JAMA Network Open*.

APLS	No	1547	408	1	-	
	Yes	19	6	1.29	(0.58 to 2.89)	0.53
Thromboembolic event	No	1460	380	1	-	
	Yes	106	34	1.33	(0.94 to 1.89)	0.11
Infection	No	1065	270	1	-	
	Yes	501	144	1.15	(0.94 to 1.41)	0.17
Pyelonephritis	No	1497	397	1	-	
	Yes	69	17	0.88	(0.54 to 1.42)	0.59
Kidney stone	No	1533	404	1	-	
	Yes	33	10	1.17	(0.63 to 2.19)	0.62
Pneumopathy	No	1504	397	1	-	
	Yes	62	17	1.04	(0.64 to 1.69)	0.87
Sepsis	No	1499	397	1	-	
	Yes	67	17	0.93	(0.58 to 1.52)	0.78
Malaria	No	1533	407	1	-	
	Yes	33	7	0.74	(0.35 to 1.57)	0.43
Bilarzia	No	1550	413	1	-	
	Yes	16	1	0.20	(0.03 to 1.45)	0.11
Tuberculosis	No	1492	385	1	-	
	Yes	74	29	1.73	(1.19 to 2.52)	0.005
Polycystic kidney	No	1415	380	1	-	
	Yes	151	34	0.82	(0.58 to 1.16)	0.26
IgA	No	1457	394	1	-	
	Yes	109	20	0.63	(0.40 to 0.99)	0.04
Glomerulopathy	No	1145	329	1	-	
	Yes	421	85	0.67	(0.53 to 0.85)	<0.001
CIN	No	1329	365	1	-	
	Yes	237	49	0.72	(0.54 to 0.97)	0.03
Vascular nephropathy	No	1497	377	1	-	
	Yes	88	37	1.94	(1.38 to 2.72)	<0.001
Sickle cell disease	No	1544	408	1	-	
	Yes	22	6	0.998	(0.45 to 2.23)	1.00
Cancer	No	1433	355	1	-	<0.001
	Yes	133	59	2.08	(1.58 to 2.74)	
Monoclonal gammapathy	No	1539	399	1	-	
	Yes	27	15	2.58	(1.54 to 4.31)	<0.001
Alcool Abuse	No	1480	389	1	-	
	Yes	86	25	1.16	(0.78 to 1.74)	0.46
Drug Abuse	No	1560	413	1	-	
	Yes	6	1	0.57	(0.08 to 4.07)	0.58
Psychiatric history	No	1477	383	1	-	
	Yes	89	31	1.46	(1.02 to 2.11)	0.04
Cognitive Disorder	No	1557	414	1	-	1.00
	Yes	9	0	-		

	Epilepsy	No	1544	410	1	-	
		Yes	22	4	0.64	(0.24 to 1.72)	0.38
	Lupus	No	1532	406	1	-	
		Yes	34	8	0.90	(0.45 to 1.82)	0.78
	Left Ventricular Hypertrophy	No	1316	337	1	-	0.08
		Yes	250	77	1.24	(0.97 to 1.59)	
	Cardiac Atrial Disorder	No	1490	368	1		
		Yes	76	46	3.33	(2.45 to 4.52)	<0.001
	Valvulopathy	No	1505	388	1	-	
		Yes	61	26	1.87	(1.26 to 2.78)	0.002
	Mechanical valv	No	1551	406	1	-	0.01
		Yes	15	8	2.46	(1.22 to 4.95)	
	Anticoagulation	No	1488	377	1	-	
		Yes	78	37	2.21	(1.58 to 3.10)	<0.001
	Peptic Ulcer	No	1469	378	1	-	
		Yes	97	36	1.54	(1.10 to 2.17)	0.01
	Gastritis	No	1497	395	1	-	
		Yes	69	19	1.07	(0.67 to 1.69)	0.78
	Diverticulosis	No	1514	391	1	-	
		Yes	52	23	1.99	(1.31 to 3.03)	0.001
	Liver disease	No	1531	404	1	-	
		Yes	35	10	1.16	(0.62 to 2.17)	0.65
	Acute pancreatitis	No	1542	408	1	-	
		Yes	24	6	0.96	(0.43 to 2.16)	0.93
	Sarcoidosis	No	1559	411	1	-	
		Yes	7	3	1.86	(0.60 to 5.78)	0.29
	Thyroid disorder	No	1522	404	1	-	
		Yes	44	10	0.88	(0.47 to 1.64)	0.68
	Ankylosing spondylitis	No	1562	412	1	-	
		Yes	4	2	3.25	(0.81 to 13.05)	0.10
	Number of drugs		1566	414	1.14	(1.10 to 1.17)	<0.001
	Previous transplantation (other organ)	No	1542	408	1	-	0.87
		Yes	24	6	0.93	(0.42 to 2.09)	
**Imaging**	Left Ventricular Mass		1566	414	1.01	(1.01 to 1.01)	<0.001
	Kidney Size		1566	414	0.81	(0.73 to 0.90)	<0.001
	Ratio kidney size/donor BMI		1566	414	0.74	(0.66 to 0.84)	<0.001
**Biology**	Creatinin		1566	414	0.999	(0.999 to 0.999)	<0.001
	Urea		1566	414	0.98	(0.97 to 0.99)	0.001
	Na+		1566	414	0.96	(0.93 to 0.99)	0.01
	K+		1566	414	0.90	(0.79 to 1.03)	0.12
	Cl-		1566	414	0.98	(0.86 to 1.00)	0.05
	Mg++		1566	414	0.47	(0.24 to 0.91)	0.03

© 2026 Debiais-Deschamps C et al. *JAMA Network Open*.

HCO3-		1566	414	0.99	(0.96 to 1.01)	0.26
Glucose		1566	414	1.05	(1.03 to 1.08)	<0.001
Glycated Haemoglobin		1566	414	1.42	(1.33 to 1.52)	<0.001
Calcium		1566	414	0.95	(0.62 to 1.48)	0.83
Phophorus		1566	414	0.79	(0.64 to 0.99)	0.02
PTH (log)		1566	414	0.92	(0.84 to 1.004)	0.06
Protein		1566	414	0.99	(0.98 to 1.003)	0.15
Albumin		1566	414	0.96	(0.94 to 0.98)	<0.001
Gamma-globulin		1566	414	0.996	(0.97 to 1.03)	0.02
Uric acid		1566	414	0.999	(0.99 to 1.00)	0.03
25-OH-Vitamin-D		1566	414	1.007	(1.001 to 1.01)	0.01
1-25-OH-Vitamin-D		1566	414	0.999	(0.99 to 1.003)	0.60
TGO		1566	414	1.008	(1.002 to 1.01)	<0.001
TGP (log)		1566	414	1.15	(0.96 to 1.37)	0.13
Gamma-GT (log)		1566	414	1.66	(1.48 to 1.85)	<0.001
PAL	≤64	1566	414	1	-	0.002
	>64			1.57	(1.16 to 1.86)	
Bilirubin		1566	414	1.01	(0.99 to 1.04)	0.35
LDH		1566	414	1.00	(0.999 to 1.001)	0.50
Total cholesterol		1566	414	0.98	(0.90 to 1.06)	0.56
HDLc		1566	414	1.035	(0.83 to 1.28)	0.76
LDLc		1566	414	0.89	(0.80 to 1.01)	0.06
Triglycerids		1566	414	0.99	(0.92 to 1.07)	0.87
Troponin	≤0.05	1383	341	1	-	<0.001
	>0.05	183	73	1.79	(1.39 to 2.31)	
CRP	≤6	1054	234	1	-	<0.001
	>6	512	180	1.70	(1.40 to 2.06)	
Iron	Q1	480	119	1	-	-
	Q2	310	96	1.26	(0.96 to 1.65)	0.09
	Q3	400	105	1.05	(0.81 to 1.36)	0.72
	Q4	376	94	1.02	(0.78 to 1.34)	0.90
Ferritin (log)		1566	414	1.22	(1.09 to 1.37)	<0.001
TP		1566	414	0.99	(0.98 to 0.99)	0.004
APTT		1566	414	0.82	(0.57 to 1.19)	0.1
Haemoglobin		1566	414	1.03	(0.97 to 1.11)	0.26
Lymphocyte count		1566	414	0.85	(0.72 to 0.99)	0.04
Neutrophile count		1566	414	1.04	(0.99 to 1.09)	0.11

The following variables were excluded from subsequent multivariable analysis: creatinine and urea due to missing information on the timing of the last dialysis session before measurement, TP due to redundancy with clinical characteristics, smoking status, glucose (cor = 0.54 with HbA1c), protein and gammaglobulin (cor = 0.34 and 0.12 with albumin, respectively), chloride (cor = 0.43 with sodium), TGO (cor = 0.25 with gGT), and PAL (cor = 0.36 with PTH).

APLS: Antiphospholipid Antibody Syndrome; APTT: Activated Partial Thromboplastin Time; BMI: Body Mass Index; Ca++: Calcium, CIN: Chronic Interstitial Nephropathy; Cl-: Chloride; COPD: Chronic Obstructive Pulmonary Disease; CRP: C-reactive Protein; DSA: Donor Specific Antibodies; ESRD: End Stage Renal

© 2026 Debiais-Deschamps C et al. *JAMA Network Open*.

Disease; gGT: gammaGlutamylTransferase; HbA1c: Glycated Haemoglobin; HBV: Hepatitis B Virus (HBV infection past or present, excluding vaccination only); HCO3-: Bicarbonate; HCV: Hepatitis C Virus; HCO3-: Bicarbonate; HDLc: High Density Lipoprotein Cholesterol; HIV: Human Immunodeficiency Virus; K+: Potassium; MACCE: Major Cardio-Vascular Event, combined criteria including coronaropathy, myocardial infarction and stroke; PKD: Polycystic Kidney Disease; LDH: Lactate Deshydrogenase; LDLc: Low Density Lipoprotein Cholesterol; Mg++: Magnesium; Na+: Sodium; PAD: Peripheral Arterial Disease; PAL: Alkaline Phosphatase; PTH: Parathyroid Hormone; TGO: Glutamic-oxaloacetic transaminase; TGP: Glutamic pyruvic transaminase; TP: Prothrombin Time.

eTable 7. Selected variables with LASSO penalized Cox model

A total of 11 variables were selected with LASSO: recipient age, dialysis duration, diabetes mellitus, major cardio-vascular events, left ventricular mass, atrial rhythm disorder, hbA1c, gGT, CRP, troponin and alkaline phosphatase.

Variable	Number of times the variable was selected over the 100 repetitions
**Recipient age**	100
**Diabetes**	100
**HbA1C**	100
**gGT**	100
**MACCE**	100
**Left ventricular mass**	99
**Atrial rhythm disorder**	99
**CRP**	95
**Troponin**	90
**Dialysis duration**	74
**Alkaline phosphatase**	74

The 10-year discrimination of the Cox model including the aforementioned variables in the derivation cohort was 0.782.

eTable 8. Selected variables with Elastic Net penalized Cox model

Elastic Net selection resulted in 16 variables: recipient age, dialysis duration, diabetes mellitus, major cardiovascular events, left ventricular mass, atrial rhythm disorder, COPD, hbA1c, gGT, CRP, troponin, alkaline phosphatase, living or deceased donation, donor cause of death, donor hypertension, and kidney size.

Variable	Number of times the variable was selected over the 100 repetitions
**Recipient age**	100
**Diabetes**	100
**HbA1C**	100
**gGT**	100
**MACCE**	100
**Left ventricular mass**	100

© 2026 Debiais-Deschamps C et al. *JAMA Network Open*.

**Atrial rhythm disorder**	100
**CRP**	99
**Donor cause of death**	97
**Troponin**	89
**Kidney transplant size**	79
**Albumin**	73
**COPD**	72
**Donor hypertension**	64
**Alkaline phosphatase**	64
**Living donation**	63

The 10-year discrimination of the Cox model including the aforementioned variables in the derivation cohort was 0.787.

© 2026 Debiais-Deschamps C et al. *JAMA Network Open*.

eTable 9. Time-dependent discrimination of the mBox in the derivation cohort

Prediction horizon	C-statistic (95% CI)
1-year	0.82 (0.77-0.87)
2-year	0.80 (0.75-0.84)
3-year	0.79 (0.76-0.83)
4-year	0.80 (0.76-0.83)
5-year	0.80 (0.77-0.82)
6-year	0.79 (0.77-0.82)
7-year	0.79 (0.76-0.81)
8-year	0.79 (0.77-0.81)
9-year	0.80 (0.78-0.82)
10-year	0.80 (0.78-0.82)

eTable 10. Calibration and overall accuracy of the mBox in the derivation cohort (10-year prediction horizon)

Calibration slope (95%CI)	Brier Score (95% CI)
1 (0.90-1.10)	0.13 (0.12-0.14)

eTable 11. Performances of the abbreviated mBox models in the derivation cohort (10-year prediction horizon)

Model	C-statistic (95% CI)	Calibration slope (95%CI)	Brier Score (95% CI)
**mBox**	0.80 (0.78-0.82)	1 (0.91-1.10)	0.13 (0.12-0.14)
**Abbreviated model for French validation**	0.79 (0.77-0.81)	1 (0.90-1.10)	0.13 (0.12-0.14)
**Abbreviated model for European validation**	0.79 (0.76-0.81)	1 (0.90-1.10)	0.14 (0.13-0.15)
**Abbreviated model for US validation**	0.79 (0.77-0.81)	1 (0.91-1.10)	0.13 (0.12-0.14)
**Abbreviated model for GPUH validation**	0.79 (0.77-0.81)	1 (0.91-1.10)	0.13 (0.12-0.14)

© 2026 Debiais-Deschamps C et al. *JAMA Network Open*.

**Abbreviated model for UCSF validation**	0.79 (0.76-0.80)	1 (0.90-1.10)	0.14 (0.13-0.15)
**Abbreviated model without cardiac ultrasonography data**	0.80 (0.77-0.81)	1 (0.90-1.10)	0.13 (0.12-0.14)
**Abbreviated model without biological data**	0.78 (0.76-0.80)	1 (0.90-1.10)	0.14 (0.13-0.15)
**Abbreviated model without imaging data and albumin**	0.79 (0.77-0.81)	1 (0.90-1.10)	0.13 (0.12-0.14)
**Abbreviated model without imaging and biological data**	0.78 (0.75-0.80)	1 (0.90-1.10)	0.14 (0.13-0.15)

eTable 12. Time-dependent discrimination of the mBox in the external validation cohorts

**Prediction horizon**	**C-statistic (95% CI)**				
	**French external validation cohort**	**European external validation cohort**	**US external validation cohort**	**GPUH external validation cohort**	**UCSF external validation cohort**
1-year	0.77 (0.72-0.82)	0.72 (0.67-0.77)	0.66 (0.54-0.77)	0.79 (0.76-0.83)	0.74 (0.65-0.81)
2-year	0.76 (0.72-0.80)	0.73 (0.69-0.76)	0.70 (0.60-0.78)	0.80 (0.78-0.83)	0.70 (0.64-0.75)
3-year	0.77 (0.73-0.81)	0.74 (0.71-0.76)	0.73 (0.65-0.79)	0.79 (0.77-0.81)	0.70 (0.65-0.74)
4-year	0.77 (0.73-0.80)	0.74 (0.72-0.77)	0.75 (0.69-0.80)	0.79 (0.76-0.81)	0.70 (0.66-0.75)
5-year	0.77 (0.74-0.80)	0.74 (0.71-0.76)	0.75 (0.70-0.79)	0.79 (0.77-0.81)	0.70 (0.66-0.74)
6-year	0.76 (0.73-0.79)	0.74 (0.72-0.76)	0.75 (0.70-0.79)	0.79 (0.77-0.81)	0.71 (0.66-0.75)
7-year	0.76 (0.73-0.79)	0.74 (0.72-0.76)	0.74 (0.70-0.78)	-	-
8-year	0.76 (0.73-0.79)	0.74 (0.72-0.76)	0.74 (0.70-0.78)	-	-
9-year	0.76 (0.73-0.78)	0.74 (0.72-0.76)	-	-	-
10-year	0.76 (0.73-0.78)	0.74 (0.72-0.76)	-	-	-

© 2026 Debiais-Deschamps C et al. *JAMA Network Open*.

eTable 13. Calibration and overall accuracy of the mBox in the external validation cohorts (respectively 10-year, 7-year, and 5-year prediction horizon for France and Europe, US, and GPUH and UCSF)

	Calibration slope (95%CI)	Brier Score (95% CI)
**French external validation cohort**	0.76 (0.66-0.86)	0.15 (0.13-0.17)
**European external validation cohort**	0.88 (0.81-0.95)	0.18 (0.17-0.19)
**US external validation cohort**	0.77 (0.60-0.93)	0.13 (0.11-0.15)
**GPUH external validation cohort**	0.82 (0.73-0.91)	0.13 (0.11-0.14)
**UCSF external validation cohort**	0.70 (0.54-0.85)	0.11 (0.10-0.13)

eTable 14. Multivariable model including recipient's sex

Predictors		Number of patients	Number of events	HR (95% CI)	p-value
Recipient's age at transplantation (years)		1,566	414	1.07 (1.06-1.08)	<0.001
Male sex		1,566	414	0.82 (0.65-1.01)	0.052
Major adverse cardio-vascular events		1,566	414	1.75 (1.41-2.18)	<0.001
Atrial rhythm disorder		1,566	414	1.47 (1.08-2.03)	0.02
Valvulopathy		1,566	414	1.51 (1.01-2.26)	0.045
Psychiatric disorder		1,566	414	2.11 (1.45-3.08)	<0.001
HCV serology		1566	414	1.53 (1.09-2.14)	0.01
Left Ventricular Mass		1,566	414	1.01 (1.004-1.01)	<0.001
Kidney transplant size/donor BMI		1,566	414	0.81 (0.72-0.92)	0.001
Dialysis time (years)	0	292	50	1	
	<3	450	123	1.18 (0.84-1.65)	
	≥3	824	241	1.54 (1.13-2.10)	0.006
HbA1c		1,566	414	1.21 (1.12-1.32)	<0.001
Albumin		1,566	414	0.98 (0.96-0.99)	0.02
Troponin	≤0.05		341	1	
	>0.05		73	1.38 (1.04-1.56)	0.02
CRP	≤6		234	1	
	>6		180	1.27 (1.04-1.56)	0.02
gGT (log)		1,566	414	1.40 (1.22-1.59)	<0.001

© 2026 Debiais-Deschamps C et al. *JAMA Network Open*.

eTable 15. Performances of the mBox with and without recipient's sex (10-year prediction horizon)

	C-statistic (95%CI)	Brier Score (95% CI)
**mBox**	0.80 (0.78-0.82)	0.13 (0.12-0.14)
**mBox with recipient's sex**	0.80 (0.78-0.82)	0.13 (0.12-0.14)

eTable 16. Discrimination of previously published mortality prediction models applied to the derivation and validation cohorts

Previously published mortality scores were applied in the derivation cohort and discrimination was evaluated at the corresponding time horizon.

For the external validation cohorts, the models were applied when possible, i.e., when the required parameters were available. To maximize the available information, patients from the validation cohorts were pooled. For the RRS, EPTS, and KTOP scores, this resulted in 6,363, 6,352, and 6,348 patients, respectively, from the Saint-Louis, Bichat, Toulouse, Tours, Leiden, Liege, Leuven, Mayo, and Philadelphia centers. For the ORLY score, 1,710 patients from the Saint-Louis, Bichat, Mayo, and Philadelphia centers were included. The Charlson Comorbidity Index, iChoose Kidney, the Transplant Score, and the model developed by Kasiske et al. could not be applied to patients in our validation cohorts because the required parameters were not collected as part of routine clinical practice.

© 2026 Debiais-Deschamps C et al. *JAMA Network Open*.

	Discrimination					
	Derivation cohort					Pooled validation cohort
	1 year	3 years	5 years	8 years	10 years	
**mBox**	0.818	0.795	0.797	0.792	0.798	-
**Charlson Comorbidity Index**	-	-	-	-	0.745	-
**RRS**	0.769	0.748	0.753	0.751	0.759	0.733 (10 years)
**EPTS**	0.769	0.752	0.754	0.750	0.757	0.737
**KTOP**	0.761	0.754	0.759	0.751	0.754	0.729 (10 years)
**Score developed by Kasiske et al.***	0.767	0.745	0.738	0.723	0.725	-
**iChoose Kidney**	-	0.768	-	-	-	-
**Transplant Score**	0.786	0.766	0.769	0.761	0.767	-
**Orly score**	0.731	0.727	0.735	0.735	0.740	0.741 (5 years)

*Two parameters were not available in our derivation cohort: insurance status and ethnicity. As health insurance is universal in France and ethnicity-based statistics are not permitted, all patients were assigned to the reference group.

© 2026 Debiais-Deschamps C et al. *JAMA Network Open*.

eTable 17. Performances of the machine learning models (10-year prediction horizon)

Machine Learning model	C-statistic (95% CI)	Brier Score (95% CI)
**Random Survival Forest**	0.78 (0.74-0.82)	0.14 (0.13-0.16)
**Random Survival Forest with Extremely Randomized Trees**	0.77 (0.73-0.81)	0.14 (0.12-0.16)
**Random Survival Forest with Maximally Selected Rank Statistics**	0.78 (0.73-0.81)	0.15 (0.13-0.16)
**Gradient Boosting with Cox model base learner**	0.78 (0.75-0.82)	0.14 (0.12-0.16)
**Gradient Boosting with decision tree base learner**	0.78 (0.74-0.81)	0.14 (0.12-0.16)
**Extreme Gradient Boosting (XGBoost)**	0.78 (0.75-0.82)	0.14 (0.12-0.16)

© 2026 Debiais-Deschamps C et al. *JAMA Network Open*.

FIGURES

eFigure 1. Kaplan-Meier survival curves in the derivation and external validation cohorts.

Upper panel: Kaplan-Meier survival curve in the derivation cohort. Values in parentheses indicate the number of events occurring each year.

Lower panel: Kaplan-Meier survival curves in the derivation and external validation cohorts.

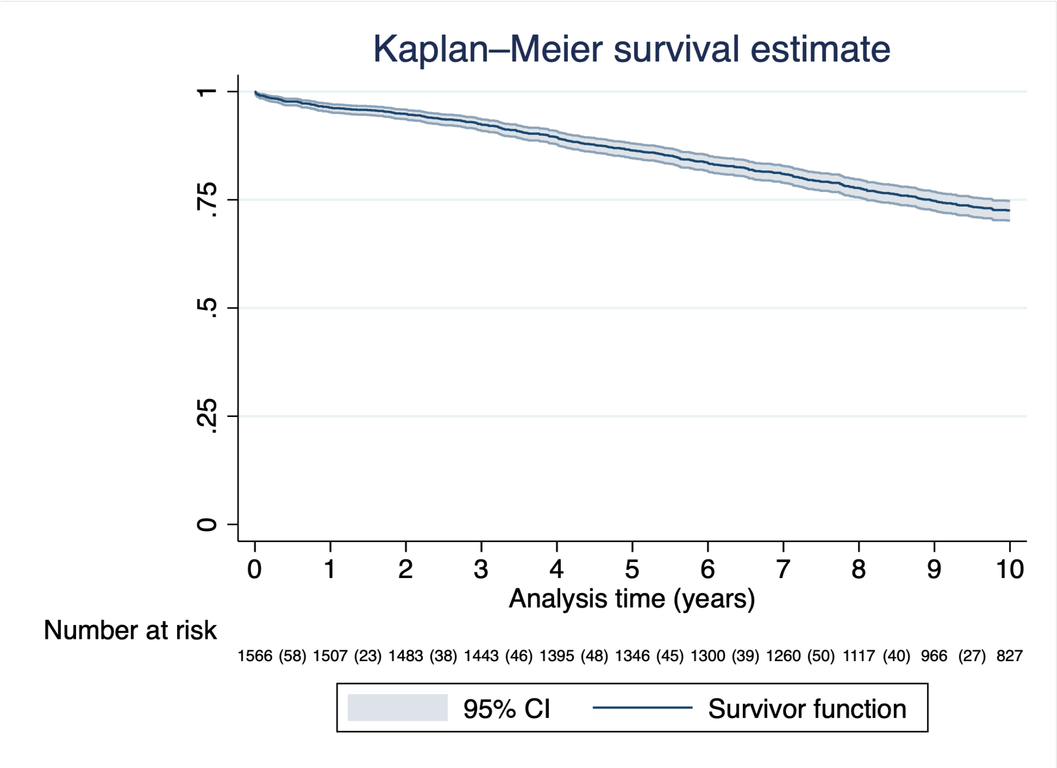

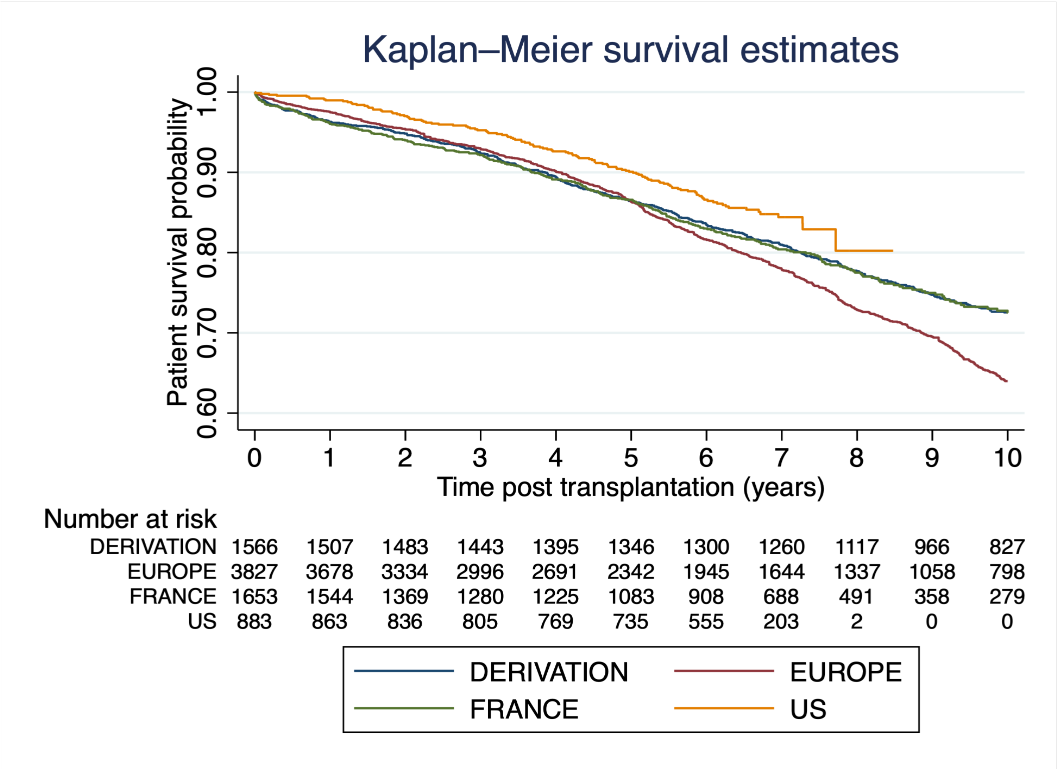

© 2026 Debiais-Deschamps C et al. *JAMA Network Open*.

eFigure 2. Distribution of the mBox score in the derivation cohort

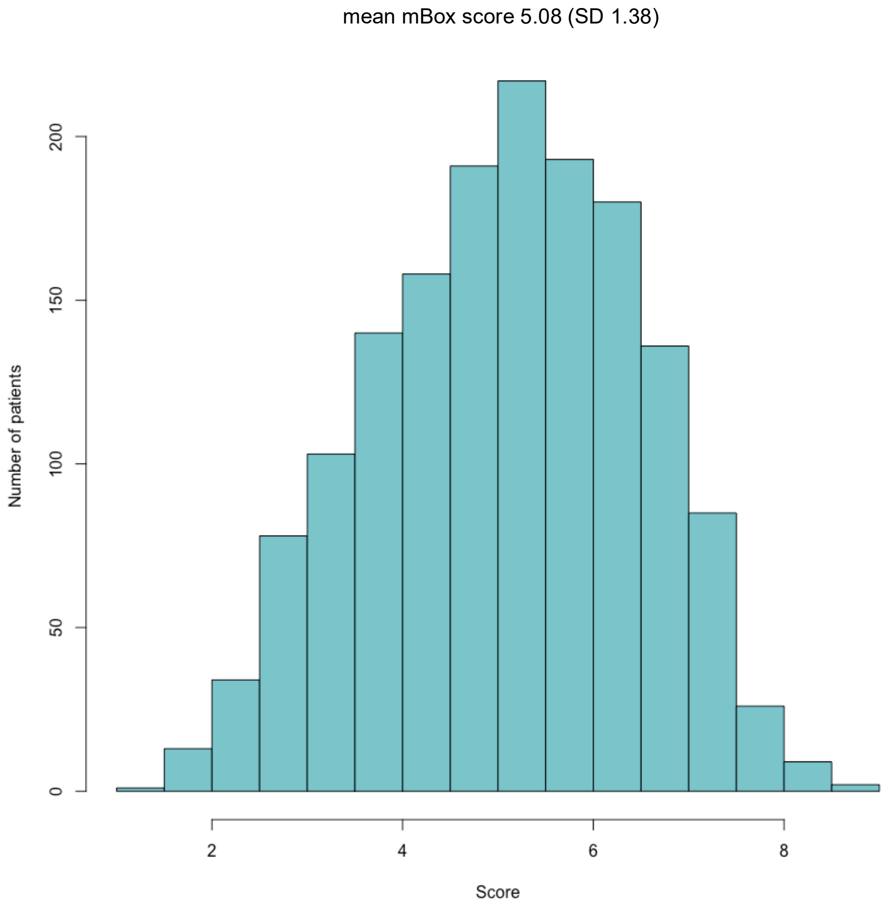

© 2026 Debiais-Deschamps C et al. *JAMA Network Open*.

eFigure 3. Calibration of the mBox score in the derivation cohort from 1 to 10 years post-transplantation

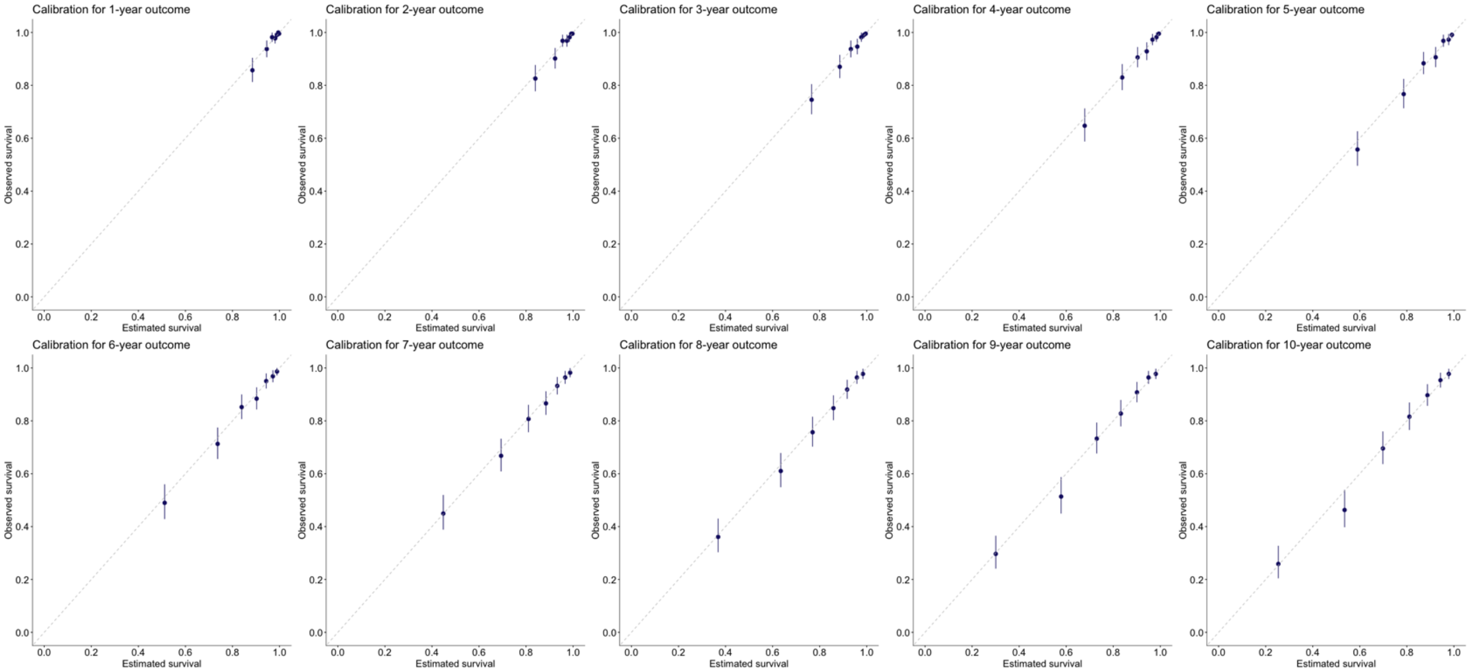

© 2026 Debiais-Deschamps C et al. *JAMA Network Open*.

eFigure 4. Decision curve analysis of the mBox in the derivation cohort (10-year prediction horizon)

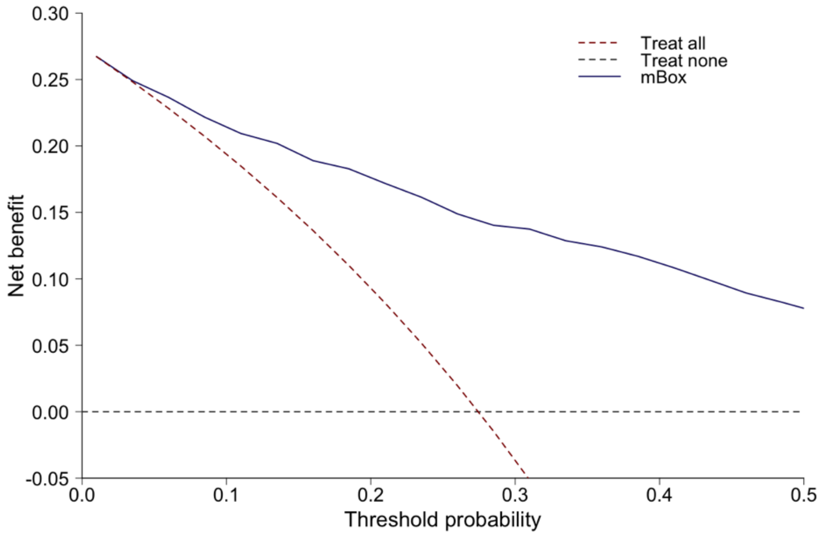

© 2026 Debiais-Deschamps C et al. *JAMA Network Open*.

eFigure 5. Calibration of the abbreviated mBox models in the derivation cohort (10-year prediction horizon)

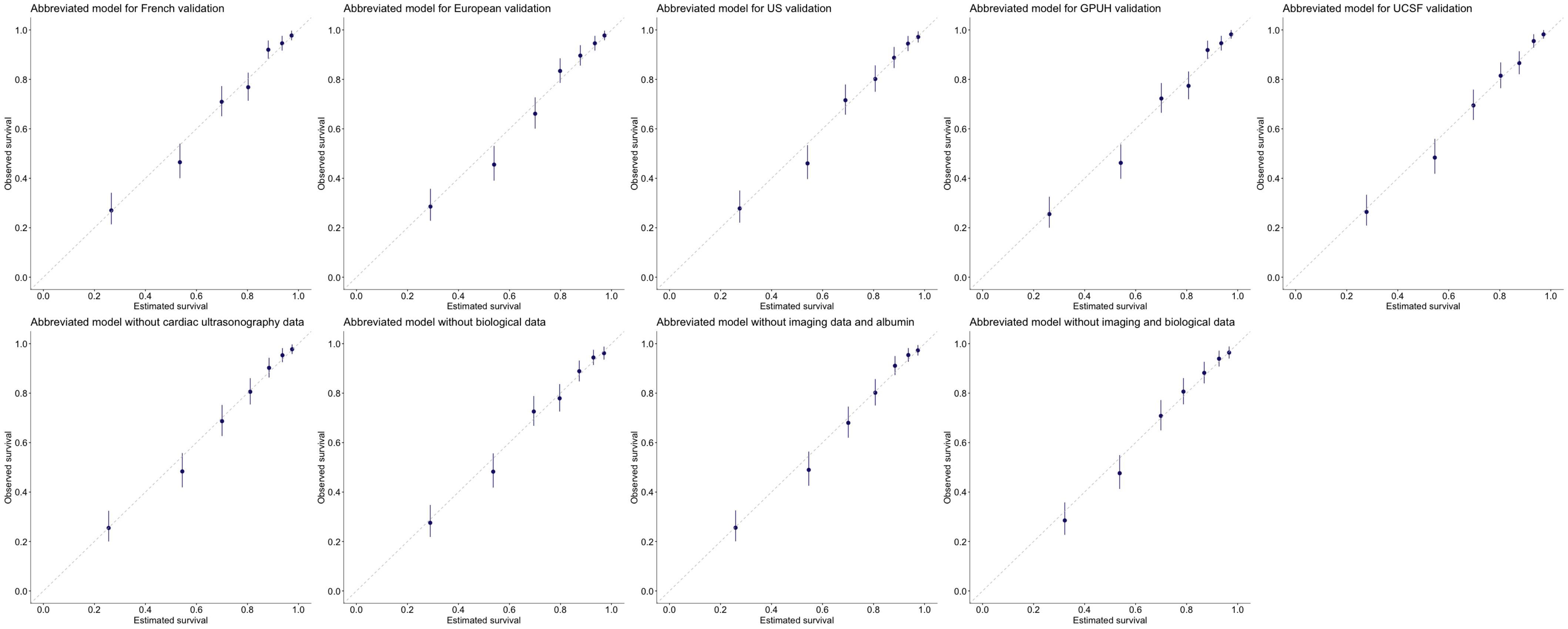

© 2026 Debiais-Deschamps C et al. *JAMA Network Open*.

eFigure 6. Decision curve analysis of the mBox and the abbreviated mBox models in the derivation cohort (10-year prediction horizon)

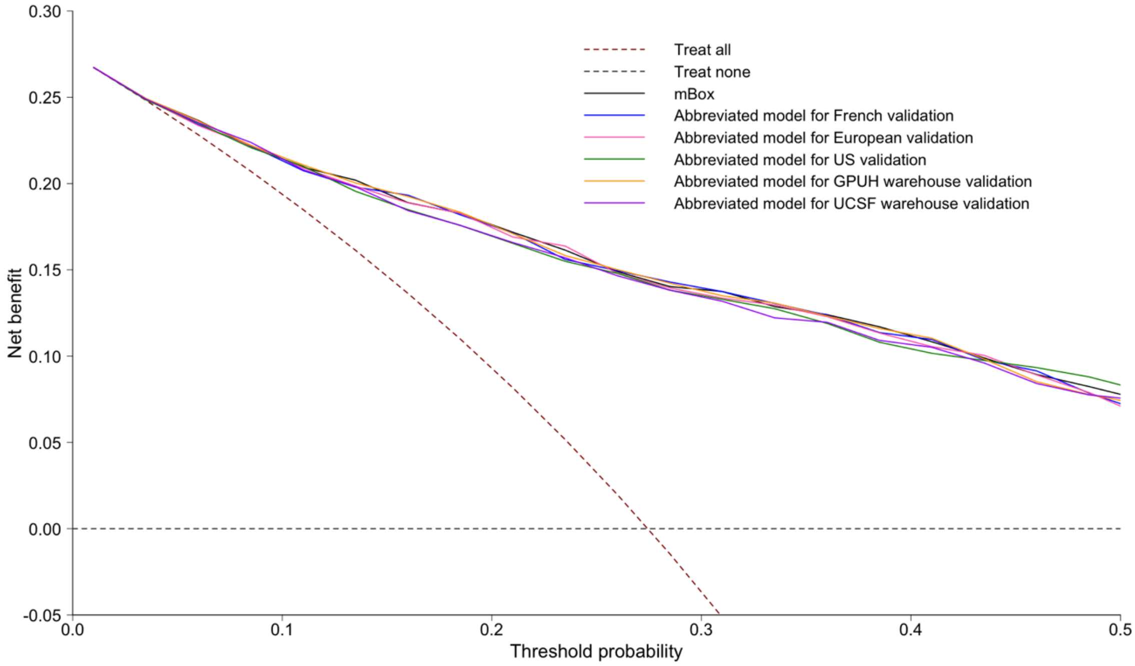

© 2026 Debiais-Deschamps C et al. *JAMA Network Open*.

eFigure 7. Calibration of the mBox score in the French external validation cohort from 1 to 10 years post-transplantation

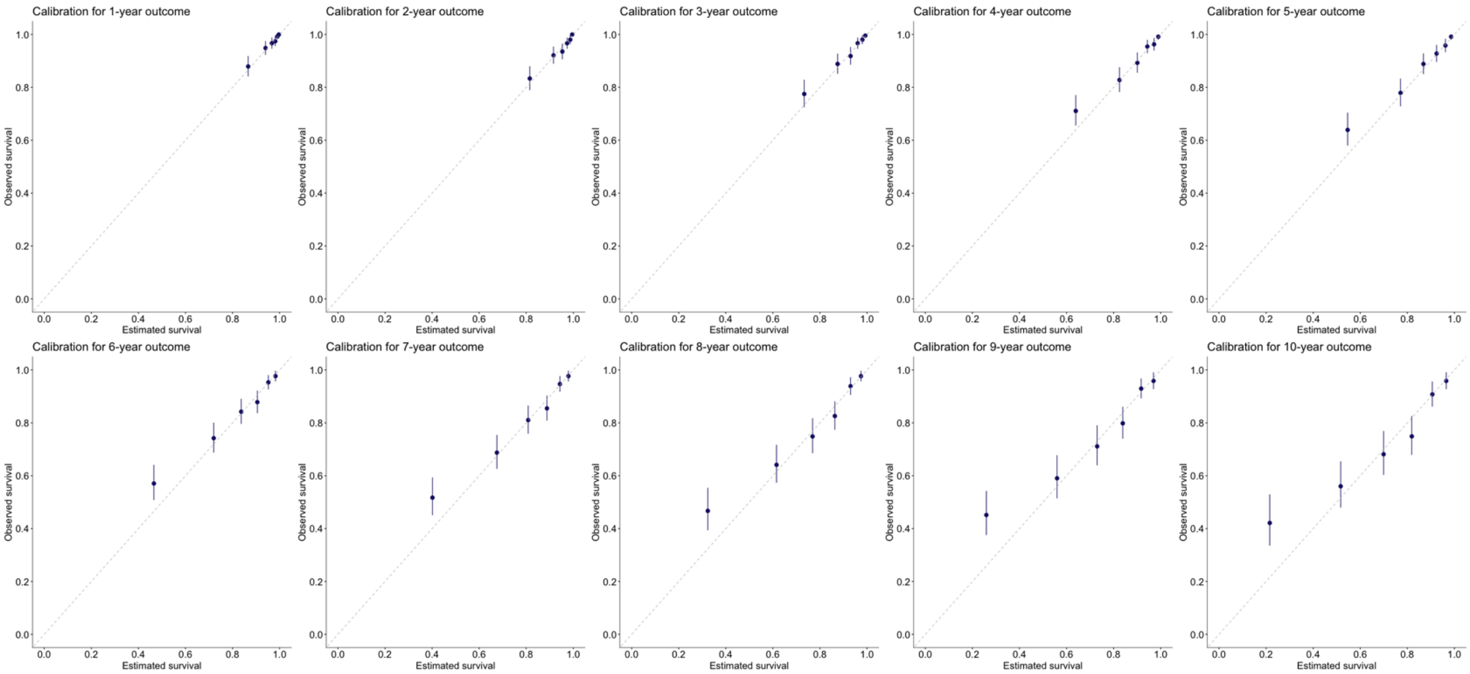

© 2026 Debiais-Deschamps C et al. *JAMA Network Open*.

eFigure 8. Calibration of the mBox score in the European external validation cohort from 1 to 10 years post-transplantation

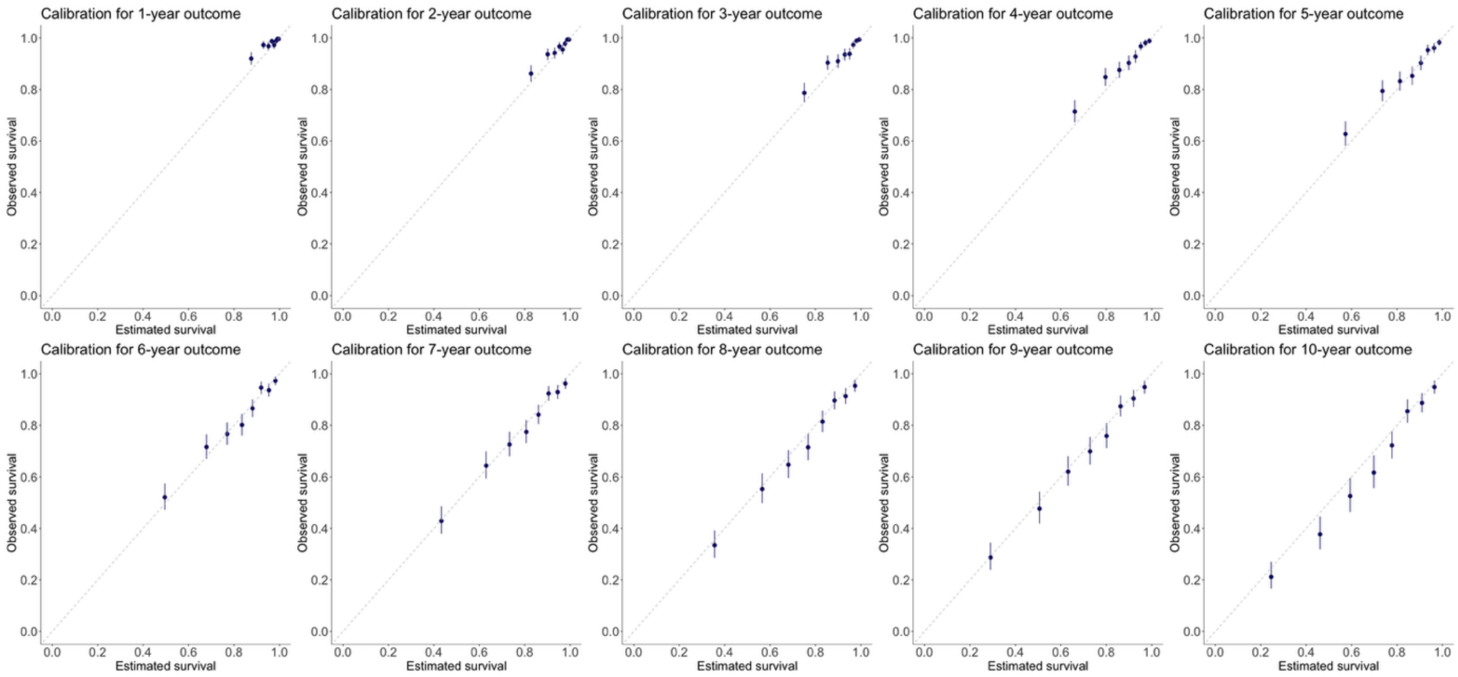

© 2026 Debiais-Deschamps C et al. *JAMA Network Open*.

eFigure 9. Calibration of the mBox score in the US external validation cohort from 1 to 7 years post-transplantation

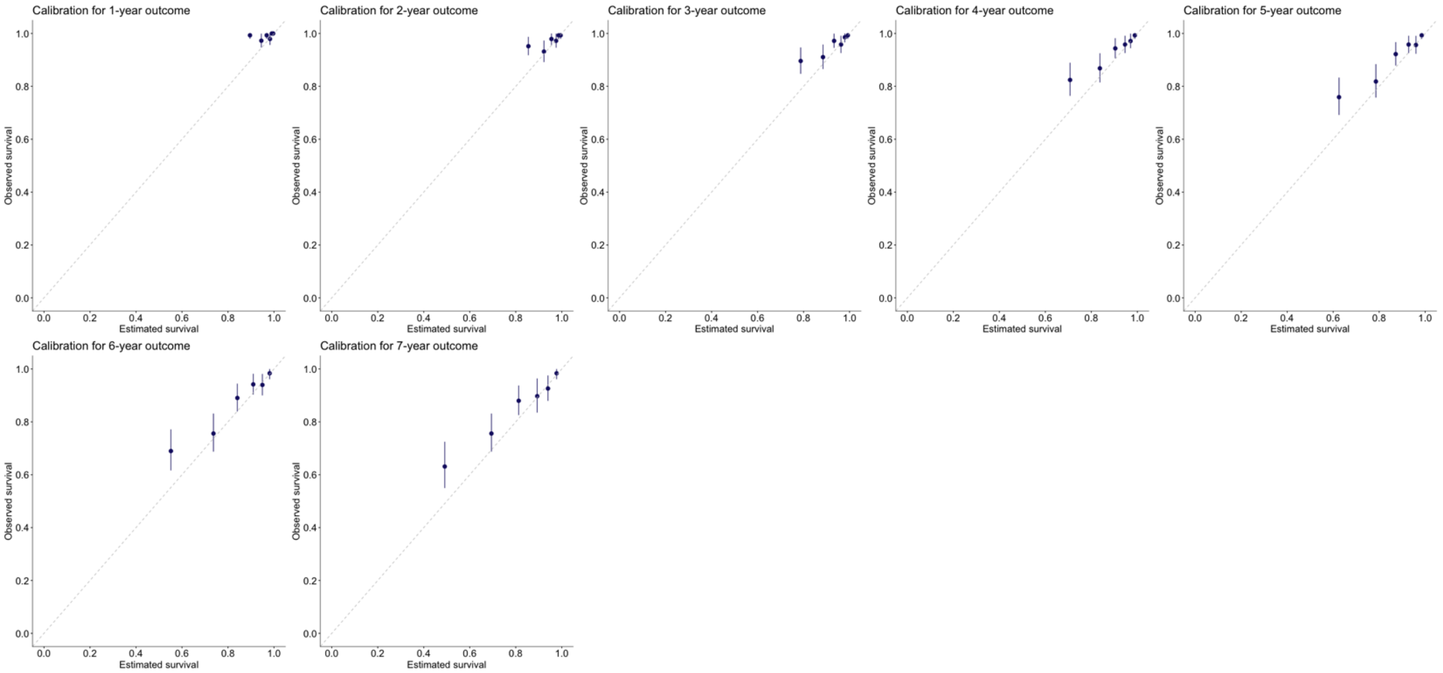

© 2026 Debiais-Deschamps C et al. *JAMA Network Open*.

eFigure 10. Calibration of the mBox score in the GPUH external validation cohort at 3 years post-transplantation

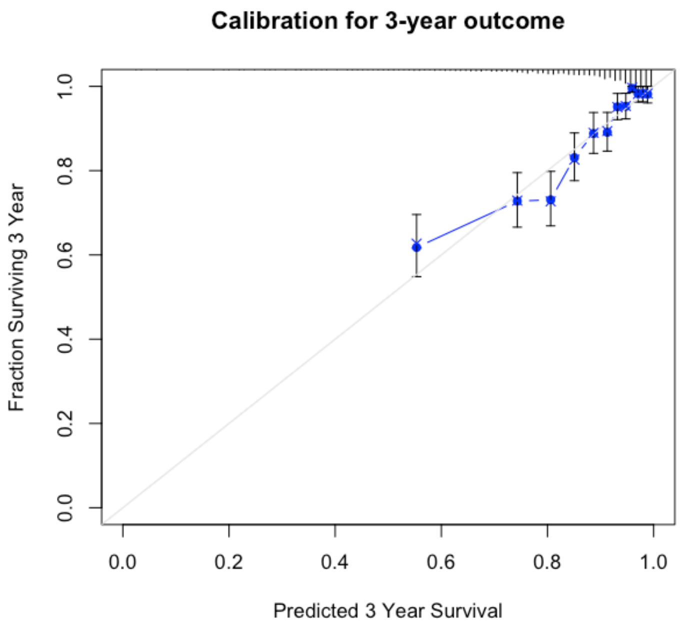

eFigure 11. Calibration of the mBox score in the UCSF external validation cohort at 3 years post-transplantation

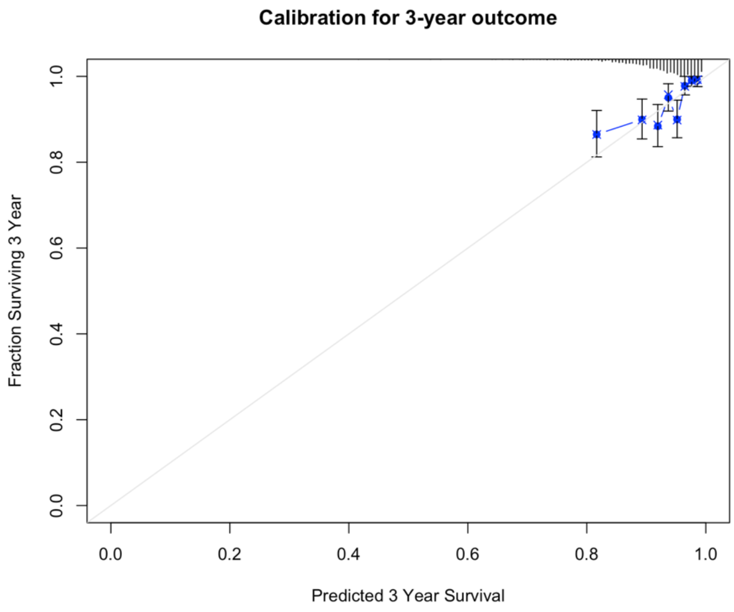

© 2026 Debiais-Deschamps C et al. *JAMA Network Open*.

eFigure 12. Decision curve analysis of the mBox in the derivation cohort (10-year prediction horizon) and in the external validation cohorts (respectively 10-year, 7-year, and 5-year prediction horizon for France and Europe, US, and GPUH and UCSF)

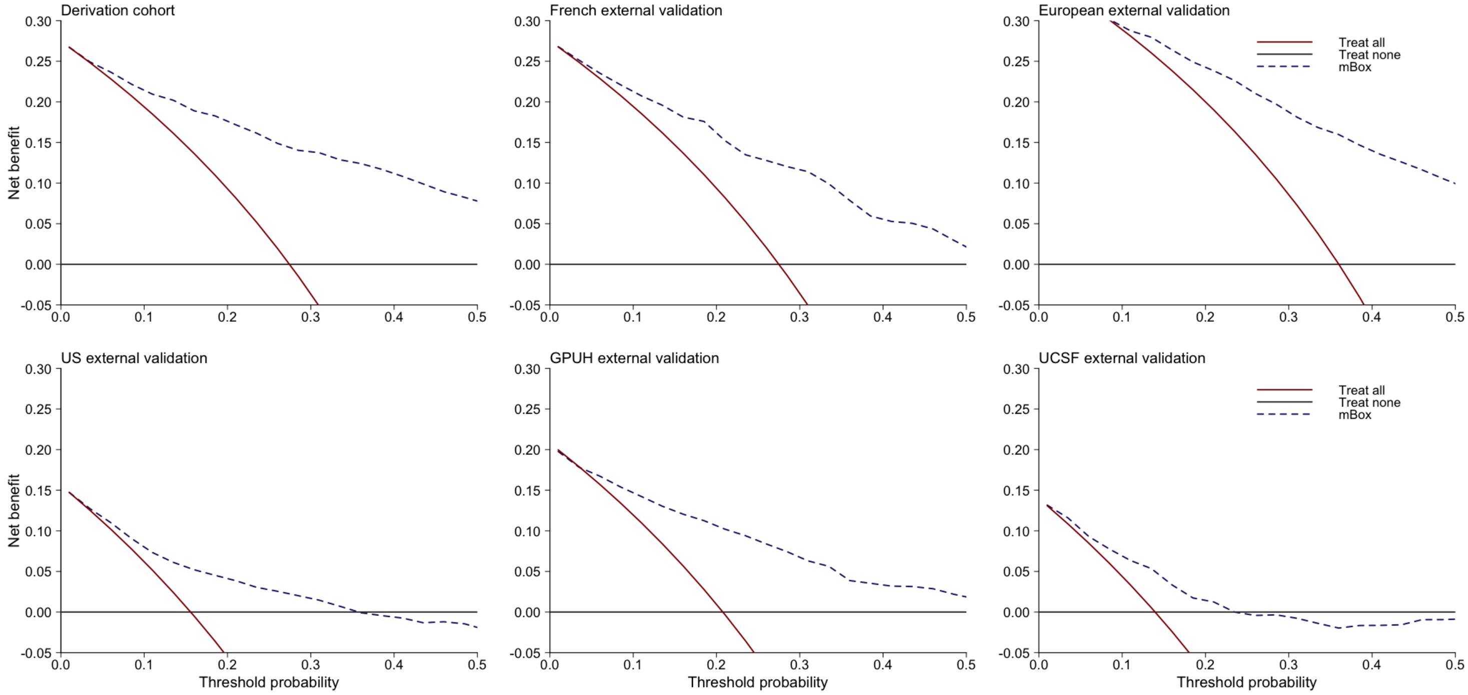

© 2026 Debiais-Deschamps C et al. *JAMA Network Open*.

eFigure 13. Calibration of the mBox and the mBox including recipient's sex as a predictor in the derivation cohort (10-year prediction horizon)

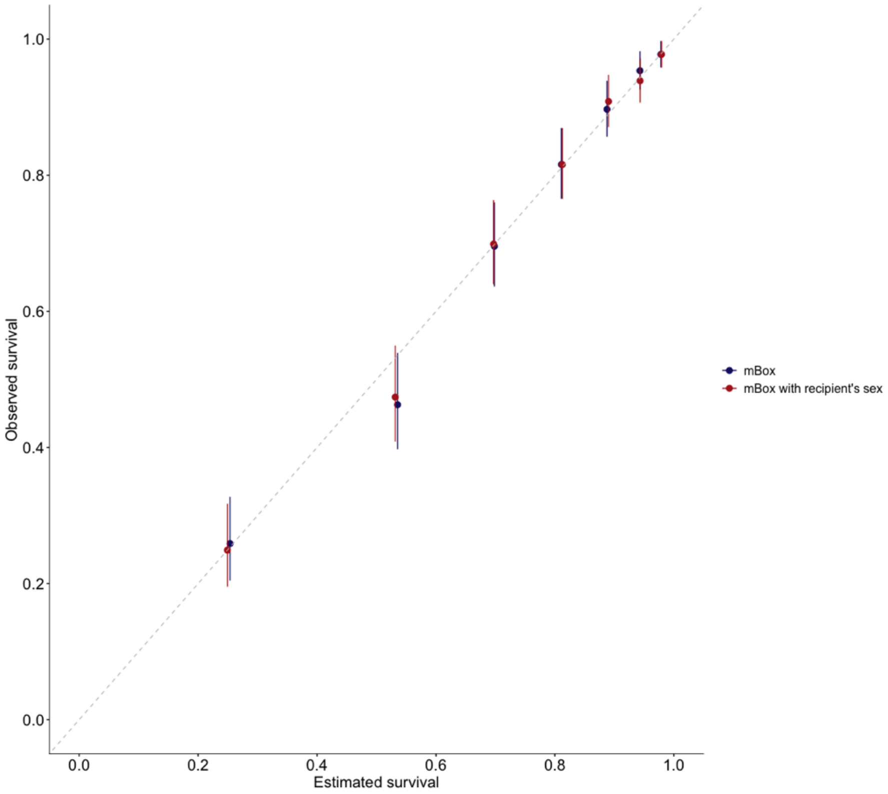

eFigure 14. Decision curve analysis of the mBox and the mBox including recipient's sex as a predictor in the derivation cohort (10-year prediction horizon)

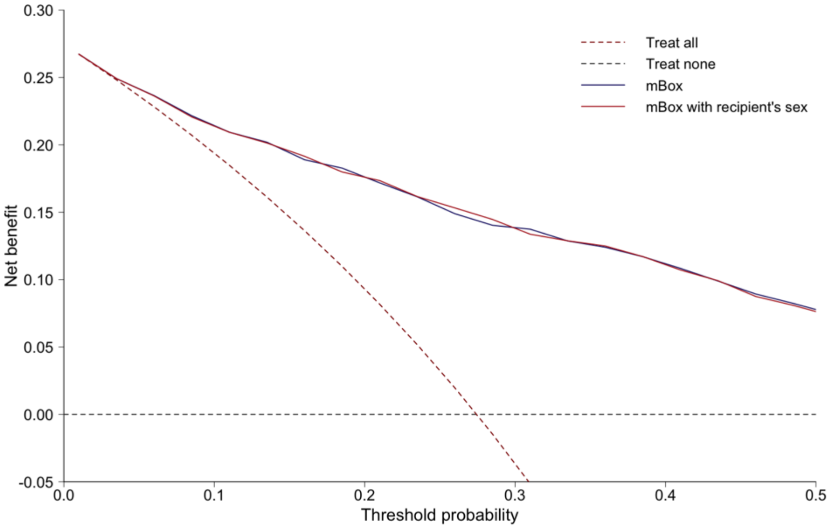

© 2026 Debiais-Deschamps C et al. *JAMA Network Open*.

eFigure 15. Variable importance of the machine learning models (top 10 most important variables)

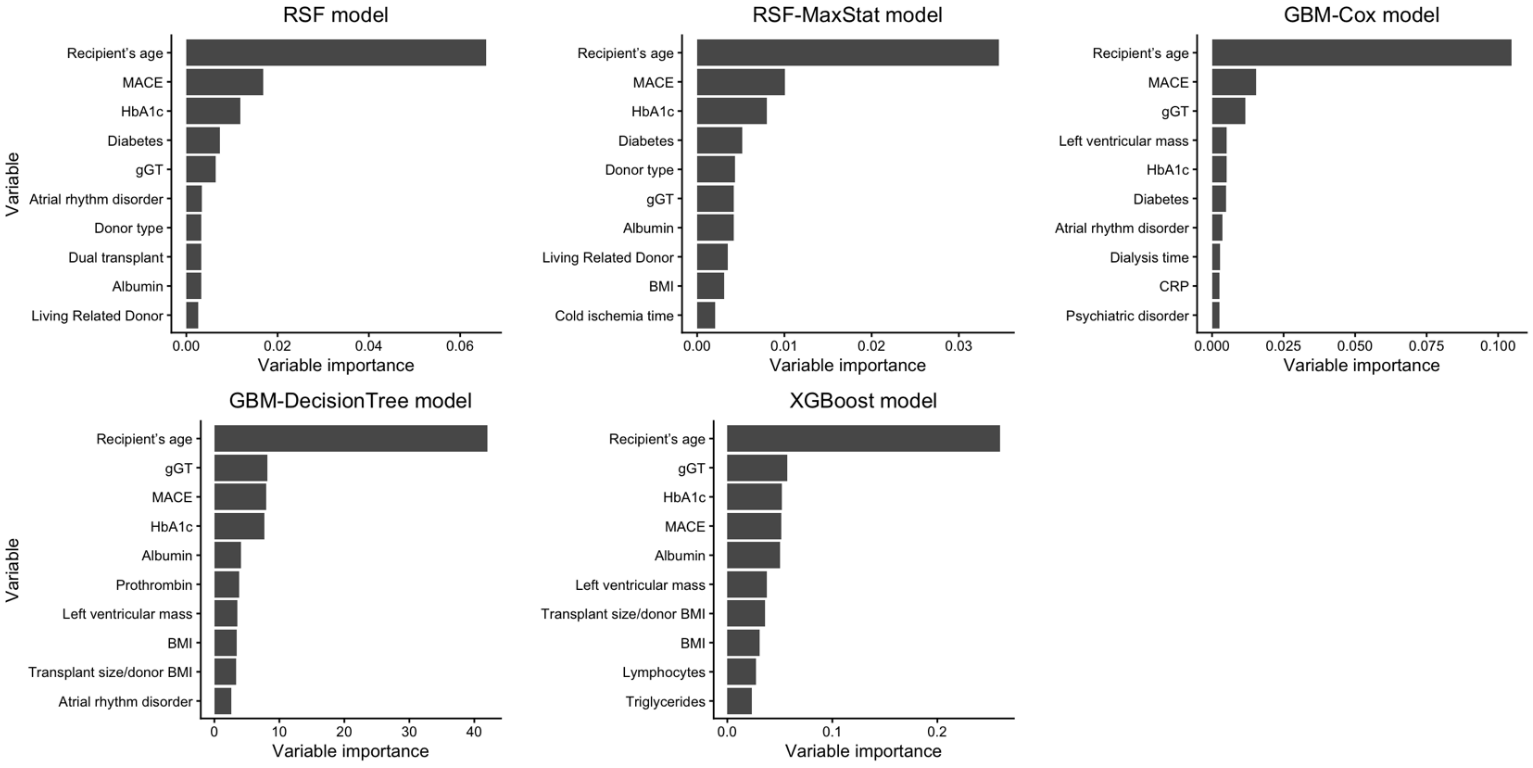

© 2026 Debiais-Deschamps C et al. *JAMA Network Open*.
