# Supplementary material for: A Prediction Model for Risk of Death in Kidney Transplant Recipients
Source: JAMA Netw Open. 2026 Apr 23;9(4):e267452. doi: 10.1001/jamanetworkopen.2026.7452 (PMC13107225; doi:10.1001/jamanetworkopen.2026.7452)
Supplement: Supplement 2. — Data Sharing Statement [file jamanetwopen-e267452-s002.pdf]

Data Sharing Statement

Debiais-Deschamps. A Prediction Model for Risk of Death in Kidney Transplant Recipients. *JAMA Netw Open*. 2026;9(4):e267452. doi:10.1001/jamanetworkopen.2026.7452

Data
Data available: Yes
Data types: Deidentified participant data
How to access data: alexandreloupy@gmail.com **When available:** With publication

Supporting Documents
Document types: None

Additional Information
Who can access the data: researchers whose proposed use of the data has been approved
Types of analyses: for external validation
Mechanisms of data availability: with a signed data access agreement
